# An Overview of Genomics, Phylogenomics and Proteomics Approaches in Ascomycota

**DOI:** 10.3390/life10120356

**Published:** 2020-12-17

**Authors:** Lucia Muggia, Claudio G. Ametrano, Katja Sterflinger, Donatella Tesei

**Affiliations:** 1 Department of Life Sciences, University of Trieste, 34127 Trieste, Italy; 2 Grainger Bioinformatics Center, Department of Science and Education, The Field Museum, Chicago, IL 60605, USA; cametrano@fieldmuseum.org; 3 Academy of Fine Arts Vienna, Institute of Natual Sciences and Technology in the Arts, 1090 Vienna, Austria; k.sterflinger@akbild.ac.at; 4 Department of Biotechnology, University of Natural Resources and Life Sciences, 1190 Vienna, Austria; donatella.tesei@boku.ac.at

**Keywords:** extremophiles, fungi, human opportunistic, lichens, plant pathogens

## Abstract

Fungi are among the most successful eukaryotes on Earth: they have evolved strategies to survive in the most diverse environments and stressful conditions and have been selected and exploited for multiple aims by humans. The characteristic features intrinsic of Fungi have required evolutionary changes and adaptations at deep molecular levels. Omics approaches, nowadays including genomics, metagenomics, phylogenomics, transcriptomics, metabolomics, and proteomics have enormously advanced the way to understand fungal diversity at diverse taxonomic levels, under changeable conditions and in still under-investigated environments. These approaches can be applied both on environmental communities and on individual organisms, either in nature or in axenic culture and have led the traditional morphology-based fungal systematic to increasingly implement molecular-based approaches. The advent of next-generation sequencing technologies was key to boost advances in fungal genomics and proteomics research. Much effort has also been directed towards the development of methodologies for optimal genomic DNA and protein extraction and separation. To date, the amount of proteomics investigations in Ascomycetes exceeds those carried out in any other fungal group. This is primarily due to the preponderance of their involvement in plant and animal diseases and multiple industrial applications, and therefore the need to understand the biological basis of the infectious process to develop mechanisms for biologic control, as well as to detect key proteins with roles in stress survival. Here we chose to present an overview as much comprehensive as possible of the major advances, mainly of the past decade, in the fields of genomics (including phylogenomics) and proteomics of Ascomycota, focusing particularly on those reporting on opportunistic pathogenic, extremophilic, polyextremotolerant and lichenized fungi. We also present a review of the mostly used genome sequencing technologies and methods for DNA sequence and protein analyses applied so far for fungi.

## 1. Introduction

The Fungi kingdom is represented by 1.5 to 5.1 million estimated species worldwide [1,2,3]. Fungi are among the most successful eukaryotes which have evolved diverse strategies even to thrive under environmental conditions where life is brought to its extremes. They have developed numerous adaptations to optimize their survival under harsh abiotic stresses [4], to colonized different substrates and to build mutualistic associations with organisms from other kingdoms (i.e., bacteria, plants and animals), thus getting advantage from the symbiotic lifestyle. Many fungi have been selected by humans for ages to be industrially exploitable organisms and are nowadays used as food or to process plant or animal materials, to produce compounds of medicinal interest or to degrade chemical compounds [5]. However, at the same time fungi can be also enemies, hardly to be defeated, as many species are serious detrimental pathogens causing economic losses to human agriculture [6], affecting animal health (human included, [7]), or damaging cultural heritages [8].

All these characteristics intrinsic of fungi require multiple changes and adaptations at deep molecular levels, which influence both the intracellular and extracellular environments. Omics approaches, nowadays including genomics, metagenomics, phylogenomics, transcriptomics, metabolomics, and proteomics have enormously advanced the way to understand fungal diversity at diverse taxonomic levels, under changeable conditions and in still under-investigated environments. These approaches can be applied both on environmental communities and on individual organism, either in nature or under in vitro conditions. In this context, cultured strains are particularly important when specific metabolic processes need to be carefully studied. However, only a minimal number of the known fungal species could be investigated for its genetic and functional diversity. Indeed, most of the taxa are difficult to retrieve in nature or even more challenging are their isolation and the stable maintenance in culture. The possibility to isolate and easily maintained certain fungal species (either yeasts or filamentous microfungi) in axenic culture is key to facilitating thoroughly researches on their genetic and metabolic traits and have led to the selection of reference models in mycology [9].

The past decade has seen the launch of uncountable -omics projects to uncover the different aspects of fungal diversity, spanning from evolution to metabolism. Large efforts have been dedicated mainly to Saccharomycotina (being *Saccaromyces cervisiae* ‘the model yeast’ within Ascomycota), to several plant pathogens responsible of serious crop infections and human opportunistic species, such as *Aspergillus*, *Fusarium* or *Cryptococcus* and *Coccidioides* to mention just a few (as reviewed in [10,11]).

Due to the huge amount of studies conducted on eumycetes, this review aims at presenting an overview of the major advances in genomics, including phylogenomics, and proteomics of ascomycetes (Ascomycota), in particular reporting on examples selected from plant and animal opportunistic and pathogenic, extremophilic/polyextremotolerant filamentous and yeast-like micromycetes, as well as lichenized fungi. We also integrated this review with notions and concepts on methodological strategies and bioinformatics tools applied for sample preparations, genome and proteome sequence data analyses, respectively.

### 1.1. Towards a Genome-Based Fungal Systematics

Fungal systematics was originally based on phenotypic characters only (i.e., macro- and micro- morphology). Although most key morphological traits are fundamental for taxonomical identification, many characters may change according to abiotic growth conditions and can lead to an unreliable classification. Thanks to technological advances, the morphology-based approach has developed into an integrative taxonomic approach based on information gained from physiology, biochemistry and molecular phylogenetics, this latter based either on DNA or protein sequence data. Molecular phylogenetics has advanced enormously in the past 20 years to improving fungal systematics independently from morphology, and the application of the phylogenetic species concept (PSC) [12] lead to the recognition of uncountable new lineages at different taxonomic levels. These studies aimed at the identification of monophyletic lineages based mainly on datasets of single or multiple loci (usually up to six loci, i.e., gene trees), and tried to include both nuclear and mitochondrial markers to improve the resolution power [13,14,15,16]. However, the preferred markers have seldom considered single copy or housekeeping genes, which indeed constitute much of the cellular genome and are essential to biological functions, providing effective markers to track organismal evolution [5]. Also, phylogenetic inferences based on different loci often revealed topological incongruences, resulting in poorly supported or unresolved clades, as each gene evolves under different evolutionary pressure and time scale (e.g., [17,18]). Instead, unlinked and randomly selected orthologous loci have reconstructed robust phylogenetic hypotheses with improved accuracy [19]. Genome sequencing has therefore become essential to deliver this amount of data and in the past few years the number of available fungal genomes grew exponentially. The use of genome-wide genetic data has led to a few new proposals on how to implement species concepts. Matute and Sepulveda [20] proposed a set of standards for using genome sequences to set species boundaries, which merge identification of reciprocal monophyly, high concordance among genomic positions, lower interspecies differentiation than intraspecific diversity and low shared polymorphisms.

However, quite a long time is usually needed to gain genomic data, as genome analyses and annotation often require the settings of several parameters in bioinformatics pipelines, particularly because most fungal genomes represent still uncharted terrains. To date (6 July 2020, NCBI), approximately 6545 fungal assemblies are publicly available, of these 5230 derive from ascomycetes and were obtained from whole-genome sequences at varying degrees of completeness. Many others have been sequenced and assembled, and wait to be released (http://genome.jgi.doe.gov/programs/fungi/index.jsf). Because of this, large scale phylogenomic studies are still relatively few while more efforts have been put on genomic analyses at species and population levels [5]. Nevertheless, the development of next-generation high-throughput sequencing is greatly accelerating the access to genomic data, which become available for phylogenomic datasets and for complementing proteomic studies, to be further integrated into fungal taxonomic and systematic studies.

### 1.2. Proteomics Advances in Mycology

In the last two decades proteomics has emerged and evolved as a powerful tool for the analysis of biological systems [21]. Investigations of the proteome encompass both the identification of the protein repertoire expressed under a given physiological state in a distinct biological space at a given time, and the assessment of changes in protein abundance in response to specific sets of conditions [22,23]. Proteomics additionally involves the study of protein posttranslational modifications and protein networks. While initially focusing prevalently on the investigation of the whole-cell proteome, with advancements in the techniques, subfields of proteomics such as secretomics, subcellular, membrane and vesicle proteomics have developed and gained a crucial role in the elucidation of protein biological functions [24]. Proteomic measurements are accomplished through a combination of highly sensitive instrumentation and powerful computational methods to produce high throughput qualitative and quantitative data. A thorough work of bioinformatic data mining plays in this respect a key role: the extraction of aggregated knowledge from the data eases the way for a better understanding of the complexities of the proteome [25]. By providing information about protein levels and pathways in a given cell or a community, proteomics data have helped shedding light on organisms’ eco-physiology and on the molecular basis of adaptive behaviours as well as the detection of protein biomarkers. Further aspects of the proteome, such as the dynamics of the protein components and the interactions among proteins and between proteins and other molecules, are deduced using proteomic tools which genomics and transcriptomics fail to offer [26].

In fungi, efforts toward post-genomic studies were initially made on a low number of widely investigated model organisms such as *Penicillium* sp., *Aspergillus* sp. and *Trichoderma* sp., along with their simpler relatives *Candida* and *Saccharomyces* sp. [27]. Since then, fungal proteomics research has progressed dramatically, especially due to the availability of powerful proteomics-based technologies and the advent of next-generation sequencing [28]. Much effort has also been directed towards the development of methodologies for the optimal protein extraction and separation, since fungal proteins are especially arduous to extract due to the chitin content of the fungal cell wall [29,30]. Protein identification has been accomplished resorting to gel-based separation techniques coupled to mass spectrometry (MS) or tandem mass spectrometry (MS/MS) and, more recently, shotgun (gel-free) methods based on liquid chromatography (LC)-MS/MS, such as bottom-up proteomics [31,32]. These approaches have paved the way for the development of databases collecting information about identity, relative abundances, localization and biological functions of proteins across a growing number of fungal species [33,34,35]. Fungal proteomics has consequently become an integral component of all “omics” sciences and systems biology approaches [28] to such an extent, that the quick generation of extraordinary amounts of data has outpaced the ability to assign functions. The growing disparity between known sequences and known functions for these proteins currently represents a unique challenge, where the availability of annotated genomic sequences plays a crucial role.

To date, the amount of proteomics investigations in Ascomycetes exceeds those carried out in any other fungal group. This is primarily due to the preponderance of their involvement in plant and animal diseases as well as to their multiple industrial applications [36]. Given the opportunistic and pathogenic nature of several species, proteomic analyses have been performed to further understand the biological basis of the infectious process [37] and to comprehend the mechanism required for the biologic control [38]. The biotechnological potential of fungal enzymes for the biosynthesis of products of significance has also driven an intense activity of proteomics research, more recently extended to the investigation of species from the extremes of life [24,39]. Several species possess excellent ability for protein production which provides one of the important aspects for identifying the protein function [40]. Furthermore, the molecular uniqueness of extremophilic and extremotolerant species has stimulated considerable interest in the search for proteins with key roles in the stress survival [41].

## 2. Genomic Advances in Ascomycota

### 2.1. Opportunistic and Pathogenic Fungi

Opportunistic fungi are usually saprobic, melanized, oligotrophic and polyextremotolerant filamentous fungi, yeasts and yeast-like micromycetes (also known as black yeasts, BY). These fungi are usually dimorphic, i.e., able of yeast-to-hyphae transition which confers them pathogenicity [42] (and references therein), and the ability to survive in extreme environmental conditions. Although the apparent lack specialized virulence traits, they can cause severe infections (chromoblastomycosis and phaeohyphomycosis) when circumstances facilitate the development of their virulence, such as presence of micro traumas on the skin caused by plant debrids, immunodeficiency and debilitation of the vertebrate hosts [7,43]. Interestingly, in the case of phaeohyphomycosis there a number of still several unexplained causes of systemic deseases in immunocompetent individuals, as some rare and peculiar neuroinfections casued by *Fonsecaea monophora* and *F. pedrosoi* [44,45,46]. Several species, in particularly *Exophiala dermatitidis*, thrive in man-made habitats, such as dishwashers, saunas, sinks and bathing facilities [47,48]. Teixeira et al. [49] sequenced and annotated a set of 23 genomes of the main human opportunistic fungi within Chaetothyriales, in the families Cyphellophoraceae and Herpotrichiellaceae, as well as related environmental species to understand their biology and divergent niche occupation with the centering aim to identifying genomic adaptations related to pathogenesis. General genomic characteristics such as transposable elements, sex-related genes, protein family evolution, genes related to protein degradation (MEROPS), carbohydrate-active enzymes (CAZymes), melanin synthesis and secondary metabolism were investigated and compared between species. In particular, a wide range of abilities of melanin biosynthesis was uncovered and genes related to metabolically distinct DHN, DOPA and pyomelanin pathways were identified, being melanin synthesis strictly connected with the pathogenicity of these fungi [48,50]. Interestingly, these melanin genes are not organized in cluster, as instead it has been observed for most fungi [51]. Alternatively, the presence of physically linked genes, forming metabolic clusters, as patterns of selections for reduced accumulation of toxic intermediate compounds (ICs) is a typical feature in the opportunistic black yeasts [51]. Also, a reduction of carbohydrate degrading enzymes was found mainly of the glycosyl hydrolase (GH) class, while most of the pectin lyase (PL) genes were lost in etiological agents of chromoblastomycosis and phaeohyphomycosis agents [49]. These fungal genomes are all similar in size, e.g., ranging from 25.8 Mb in *Capronia coronata* to 43 Mb in *Cladophialophora immunda* (CBS 834.96) and a typical, shared genomic feature seems to be the exceptionally high GC content (49–54.3%) [51]. Members of the *bantiana*-clade are among the taxa having the largest black yeast genomes, whereas species in the *dermatitidis*-clade have considerably smaller genomes. Furthermore, in these fungi the analysis of the MAting Type locus (MAT) revealed that a low selective pressure is acting at this locus, which suggests that a parasexual cycle may play an important role in generating diversity among these fungi. It was shown that members of the asexual genera *Fonsecaea* and *Cladophialophora* are heterothallic, with a single copy of either MAT-1-1 or MAT-1-2 in each individual, while all *Capronia* species were found to be homothallic, as both MAT1-1 and MAT1-2 genes were found in each single genome [49].

Relationship between opportunistic potential and polyextremotolerance instead were investigated by Gostinčar et al. [52] in a phylogenetic and a comparative genomic analysis of 20 dothideomycetous and eurotiomycetous black fungi. Comparison with genomes of specialized fungal plant pathogens revealed that known virulence-associated genes that encode secreted proteases, carbohydrate active enzyme families, polyketide synthases, and non-ribosomal peptide synthases, are signatures lacking in human opportunists but significantly enriched in the plant pathogens. These results let the authors to interpret opportunistic infections as evolutionary dead ends for these fungi which unlikely lead to true pathogenicity. Indeed, the human body seems to be neither the preferred habitat of such species, nor important for their evolutionary success, so that opportunism appears opposed to pathogenicity, where infection is instead advantageous for the species’ fitness [49]. Opportunistic fungi are generally incapable of the host-to-host transmission and therefore any host-specific adaptations would be lost with the resolution of the infection, thus explaining the observed lack of specialised virulence traits in their genomes [52].

The first sequenced genome of an opportunistic fungus was that of *Wangiella (Exophiala) dermatitidis*, the most studied opportunist species [53]. The genomic analyses revealed that *W. dermatitidis* has lost the ability to synthesize alpha-glucan, a cell wall compound that many pathogenic fungi use to evade the host immune system. On the contrary, *W. dermatitidis* contains a profile of chitin synthase genes similarly to related fungi and strongly induces genes involved in cell wall synthesis in response to pH stress. A full set of fungal light-sensing genes was also found as part of a carotenoid biosynthesis gene cluster, and a two-gene cluster involved in nucleotide sugar metabolismwas identified and characterized to have originated from a horizontal transfer event between fungi and algal viruses [53].

Very recently, Moreno et al. [54] sequenced and annotated the nuclear and the mitochondrial genomes of *Arthrocladium fulminans* (CBS 136243), the only clinical opportunistic representative of the family *Trichomeriaceae,* which otherwise is comprised of rock-inhabiting and epiphytic species of black fungi [55]. The genome of *A. fulminans* contains 315 genes coding for putative carbohydrate-active enzymes (CAZymes), which is the lowest reported number of CAZymes in *Chaetothyriales*, ranging between 339 genes in the opportunist *Exophiala dermatitidis* to 506 genes in the hydrocarbon-associated species *Exophiala xenobiotica*. Being the family Trichomeriaceae ancestral to Cyphellophoraceae and Herpotrichiellaceae, these data suggest that the increased abundance and diversification of CAZymes in chaetothyrialean black yeasts is a recent evolutionary event in Herpotrichiellaceae [54]. Further analyses of nuclear and mitochondrial orthologous genes support evidence that gene family expansion took place later in the evolution of the black yeasts and the repertory of genes associated to resistance and nutrient uptake was reduced, but not absent, in basal lineages of black yeasts [54].

Comparative genomic analysis was also undertaken for environmental and pathogenic siblings of the two genera *Fonsecaea* and *Cladophialophora*, of which some species are the etiological agents of chromoblastomycosis [56]. As for the previously mentioned opportunistic fungi, expansions of protein domains such as glyoxalases and peptidases suggested their pathogenicity, while the use of nitrogen and degradation of phenolic compounds was enriched in environmental species. The similarity of CAZymes vs. protein-degrading enzymes associated with the occurrence of virulence factors suggested a general tolerance to extreme conditions, and consequently the opportunistic tendency of *Fonsecaea* sibling species [56].

Opportunistic species of small mammals and occasionally of humans are recovered in the order Onygenales (Eurotiomycetes). Herewith, the genera *Blastomyces*, *Emmonsia*, *Histoplasma* and *Paracoccidioides* have been genome sequenced in the past years [42]. Also in these fungi, large genome sizes was detected and associated to expansion of repetitive sequences. More precisely, the genome expansion in *Blastomyces* strains resulted from a proliferation of gypsy LTR retrotransposons, which accounted for almost all repetitive DNA. Surprisingly, in *Blastomyces* genomes a bimodal distribution of GC-content organized in large isochore-like regions was also discovered. This represented the second record of a isochore-like structure of GC-regions, as it was previously detected only in *Leptosphaeria maculans* [42].

### 2.2. Plant Pathogens

Plant pathogenic fungi, which represent a major threat to agro-ecosystems and impact global food security, are among the most sequenced organisms for the study of their genomic diversity and architecture. Understanding the evolution and population dynamics of fungal plant pathogens is key to enable an appropriate management of ecosystems in modern agriculture and to contain fungal infections, that otherwise would cause severe economic losses. Uncountable studies and outstanding review compilations have been already published on several groups of fungal pathogens as well as specifically on model species—such as those belonging to the genera *Aspergillus*, *Fusarium* and *Verticillium*, to mention just a few—to investigate evolving transposable elements and virulence genes that cause genomic plasticity and variation in virulence phenotypes [11,57,58]. Pedro et al. [59] integrated genomic and phenotypic data from plant pathogen species into an expertly curated catalog of genes (PhytoPath) with experimentally verified pathogenicity, with the Ensembl tools for data visualization and analysis (www.phytopathdb.org).

Nowadays, hundreds of genomes of ascomycetous plant pathogens are available and their analyses identified chromosomes compartments distinguished into core and accessory regions in which effectors genes are located and evolve [60,61,62]. These DNA regions are either islands of repetitive DNA or entire accessory, dispensable chromosomes [61], the sequences of which are structurally variable, so that the difficulty of assembling and comparing them makes the study of their evolutionary patterns particularly challenging [63]. Lineage-specific regions have been described in plant pathogens from different subphyla such as for *Taphrina* (Taphrinomycotina), *Verticillium* and *Fusarium* ([58] and references therein). It was supposed that genomic regions with a high density of repetitive elements are hot spots for the creation of new pathogenicity-associated, lineage-specific effector genes ([58], and references therein). These latters are genes coding for small secreted proteins that enhance infection by manipulating the plant host metabolism [63]. Among the plant pathogens, biotrophic fungi were found to contain higher proportion of effector genes than hemibiotrophic and necrotrophic fungi [64]. An analysis by Lo Presti et al. [64] on 89 fungal species exhibiting different lifestyles found that genomes of saprotrophs, necrotrophs and hemibiotrophs were significantly enriched in plant cell wall degrading enzymes (PCWDE). In contrast, genomes of obligate biotrophs and symbionts were enriched in small secreted proteins [63,64]. However, the mechanisms that enabled the formation of these regions of genomic innovations remain unclear. Very recently, Potgieter et al. [65] revised the approaches of genome analyses to discovery the genetic variants that characterized the high structural variation among the accessory compartments of the effector genes. In their analyses, the authors have proposed a pipeline based on *de novo* genome assembly of short read data followed by whole genome alignment, using simulated data sets with properties mimicking that of fungal pathogen genomes.

Furthermore, genome mining of plant pathogens has also in depth investigated genes responsible for clonality or alternatively for sexual reproduction (the MAT loci) Recombination events of these genes among individuals seem to sway resistance against pesticide or host specificity, as demonstrated for *Veriticillium dahliae*, for which cryptic sexual reproduction was discovered ([58,66] and references therein).

### 2.3. Entomopathogenic Fungi

Entomopathogenic fungal species are known to infect and kill insects. They belong to the different phyla of Microsporidia, Chytridiomycota, Entomophthoromycota, Basidiomycota, and Ascomycota. In particular, ascomycetes entomopathogens falling into three families in the order Hypocreales (Cordycipitaceae, Clavicipitaceae, and Ophiocordycipitaceae) are the best studied groups for which many genome sequences are available, such as those for genera and species of *Metarhizium* spp., *Beauveria bassiana, Ophiocordyceps* spp., *Cordyceps* spp., *Tolypocladium* and *Hirsutella* ([67,68] and references therein). Because of their wide geographic distribution and extraordinary versatility, including broad lifestyle options, extremely flexible metabolism and an impressive array of colonized environments, *Metarhizium* and *Beauveria* have become refence models of entomopathogenic fungi. Species of both genera are frequently exploited as industrial sources of catalytic enzymes and as biocontrol agents. Alternatively, *Cordyceps* and *Ophiocordyceps* species are of medicinal interest and are widely used in the traditional Chines medicine, being producer of secondary bioactive metabolites against other microorganisms and cancer cells ([67,68,69,70] and references therein). Species of the genus *Metarhizium* are the best-studied entomopathogenic fungi at the molecular and biochemical levels [69,71]. Whole genome analysis of *M. anisopliae* indicated significant macrosynteny with *M. robertsii* but with some large genomic inversions, and lower sequence homology in comparison to *M. acridum*. A MAT gene analysis suggested putative homothallism in *M. anisopliae* but putative heterothallism in *M. acridum* and *M. robertsii*. Also, repetitive DNA and repeat-induced point mutation (RIP) analyses revealed *M. acridum* to have twice the repetitive content of the other two species and *M. anisopliae* to be five times more RIP affected than *M. robertsii* [72].

Genomic analyses of *Beauveria bassiana* found many species-specific virulence genes and gene family expansions and contractions which were associated to host ranges and pathogenic strategies [67]. Further comparative genomics has confirmed that secreted proteins are much more numerous in the entomopathogenic species than in the ancestral plant pathogens, highlighting the greater complexity of genes acting in the fungus-insect interactions and thereby contributing to the fungal virulence [69,73,74].

Very recently Shu et al. [69] took advantage of the single-molecule sequencing approach to sequence and assemble a highly contiguous, improved genome of *Ophiocordyceps sinensis*, while Jin et al. [74] sequenced the corresponding anamorph *Hirsutella sinensis*. Using RNA-seq and homologous protein sequences Shu et al. [69] identified over 8000 protein-coding genes and 63 secondary metabolite gene clusters, whereas Jin et al. [74] predicted and drawn the biosynthesis pathways of seven metabolites (mannitol, cordycepin, purine nucleotides, pyrimi- dine nucleotides, unsaturated fatty acid, cordyceps polysaccharide and sphingolipid) and studied the infection process and mechanism of *H. sinensis*.

### 2.4. Extremophilic/Polyextremotolerant Fungi

*Thermophilic fungi*—Thermophilic fungi are heat-tolerant species that have adapted to high temperatures, presenting growing optimal temperature higher than 40 °C; they are commonly found in composting systems [75,76]. Within the Ascomycota, thermophilic fungi evolved in at least two lineages, i.e., the classes Eurotiomycetes (in the orders Eurotiales and Onygenales) and Sordariomycetes (Sordariales, Chaetomiaceae; [77]). Because of their peculiar physiological adaptations, these fungi stand as important systems to explore as they represent potential reservoir of thermostable enzymes which can be exploited in industrial applications. However, they are rare species, often difficult to isolate and maintain in axenic culture. Therefore, genome sequencing complemented with studies of protein structures is of key importance to understand the thermal stability of the produced fungal proteins. In this context, *Chaetomium thermophilum* has become one of the first model of thermophilic fungi (it has an optimal growth temperature at 50–55 °C) for which genome sequence was obtained [78]. At the time of its complete sequencing and annotation, the genome of *C. thermophilum* was searched for genes of thermostable nucleoporines (Nups) of the nuclear pore complex (NPC) to be cloned and heterologously expressed in yeast for further industrial exploitation [78]. Nups genes are highly conserved from yeast to humans, but the mesophilic Nups are more difficult to handle than the thermostable ones produced by *C. thermophilum*. Few years later, the annotation of the *C. thermophilum* genome was considerably improved and other thousands of genes were characterized for their transcripts and expression [79].

Together with *C. thermophilum*, also *Myceliophthora thermophila* and *Thielavia terrestris* are two of the best characterized thermophilic fungi in terms of thermostable enzymes and cellulolytic activity. Fermentation characteristics of these two species have been examined and were considered suitable for large-scale production. Berka et al. [80] performed a comparative genomic analysis of these two thermophilic ascomycetes and found that their genomes both contain larger fractions of repetitive elements and have low GC content, introducing significant GC variation. When comparing the GC content of *M. thermophila* and *T. terrestris* with the mesophilic *Chaetomium globosum* and other species within Sordariomycetes, it appeared that the thermophilic species instead have a higher GC content in coding regions, which is reflected in the third codon positions. As G:C pairs are more thermally stable, it may suggest the potential adaptability of protein-coding genes to high temperatures [80]. Also, genome analyses and experimental data have suggested that both *M. thermophila* and *T. terrestris* are capable to hydrolyzing all major polysaccharides found in biomass [80].

The presence of several DNA pathways related to mechanisms of thermal adaptation, in particular a ubiquitin degradation pathway was uncovered in analyzing the genome of *Termomyces lanuginosus* (Eurotiales; generally growing on dead woody material; [81]). Ubiquitin is known to play a crucial role in the responses to various stressors, such as nutrient limitation, heat shock, and heavy metal exposure and may be essential for *T. lanuginosus* to adapt during rising temperatures in composting materials [82]. Several mechanisms of DNA condensation machinery and repairing were also genome mined and they seem to make this fungus adapted to living at high temperatures [81].

More recently, de Oliveira et al. [82] profiled the available genomes of all these thermophilic fungi and of their phylogenetically related mesophilic species for genes encoding for peptidases and their putative adaptations for thermal stability. Peptidase is one of the most important groups of industrial enzymes accounting for about 65% of the total enzyme production worldwide ([83] and references therein). A better knowledge of these thermostable enzymes and the respective coding genes would thus improve the industrial production. The authors generated an extensive catalogue of these enzymes ranging from 241 to 820 peptidase genes and found that thermophilic fungi have experienced a genome reduction in response to thermal adaptation, and consequently have lost also peptidases coding genes during their evolution. The authors [82] showed the largest reduction in the peptidase families with a higher number of genes, while those with fewer or single copies were less affected. The great amount of amino acid residues Ala, Glu, Gly, Pro, Arg and Val in peptidases from thermophilic fungi are in line with reports that some of these amino acids increase the thermal stability of the proteins [82].

*Psychropilic fungi*—Psychrophilic fungi adapted to thrive in very cold environments (i.e., in alpine, Arctic and Antarctic environments; [83]) thanks to their ability of producing antifreeze proteins (AFP) and ice-binding proteins (IBP). These proteins have evolved independently in all cold-adapted organisms, and own unique properties that attenuate the effects of intense cold and freezing on cell membranes and DNA molecules [83], bearing potentials for biotechnological applications in several disciplines [84]. Psychrophilic fungi represent therefore important, fascinating sources of AFP and IBP and, similarly to thermophilic fungi, are targeted by multiple -omics approaches.

The genus *Pseudogymnoascus* in the family Pseudorotiaceae (Thelebolales) includes extremophilic fungi known to be either aggressive pathogens (i.e., *P. destructans* pathogen for bats) or soil inhabiting psychrophilic and halotolerant taxa; many of them were isolated from permafrost whereas others from temperate soils [85,86]. Genotypes of 14 strains of *Pseudogymnoascus* were genome sequenced to shed light on recombination events, horizontal gene transfers and parasexual processes that were noted to happen among the species [86]. All sequenced strains were haploid and revealed high nucleotide diversity, with an evolutionary distance at synonymous sites which suggested that the last common ancestor of these strains lived at least 50 Mya. The strain from permafrost did not form a separate clade but had close relatives from temperate environments. Certain short genomic segments revealed phylogenetic patterns strikingly different from the rest of the genome. In particular, the MAT locus was found to have a heterothallic configuration in all strains and its two distinct alleles were detected to be interspersed along the whole-genome phylogenetic tree, resembling of multiple genetic transfer events [86].

The mitochondrial genome of the congeneric cold-adapted *Pseudogymnoascus pannorum* was sequenced [83] and consists of 13 standard protein-coding genes, two ribosomal RNA subunits and 27 transfer RNAs. The phylogenetic analysis performed on concatenated protein sequences supported *P. pannorum* as a Leotiomycetes species closely related to *P. destructans*, as supported by nuclear genes [87].

Comparative genomics analysis of the obligate psychrophilic species *Mrakia psychrophila* indicated that it has a specific codon usage preference especially for codons of Gly and Arg, and its major facilitator superfamily (MFS) transporter gene family underwent expansion [88]. The complementing transcriptomic analysis revealed that genes involved in ribosome and energy metabolism were upregulated at 4, while genes involved in unfolded protein binding, protein processing in the endoplasmic reticulum, proteasome, spliceosome, and mRNA surveillance were upregulated at 20. As in other psychrophiles, desaturase and glycerol 3-phosphate dehydrogenase, which are involved in biosynthesis of unsaturated fatty acid and glycerol respectively, were also upregulated at 4 in *M. psychrophila*. Cold adaptation of this species was explained by the synthesis of unsaturated fatty acids to maintain membrane fluidity and accumulating glycerol as a cryoprotectant [88].

A few years ago, a novel psychrophilic, endemic fungus was described for Antarctica, *Antarctomyces pellizariae* (Thelebolales, Leotiomycetes, Ascomycota; [89]). Recently, both its nuclear and mitochondrial genomes were mined in search of secondary metabolite gene clusters and genes coding for AFP and IBP proteins [89]. Within the mitochondrial genome the gene arrangement was found in line with that of other 16 Leotiomycetes and four *Pseudogymnoascus* species. The only difference was the localization of the cob gene, that was found between cox2 and cox1 genes. A putative IBP of 236 amino acids that shared sequence similarities with two IBP isoforms from the closely related *A. psychrotrophicus* was identified. Such discovery confirmed these two *Antarctomyces* species the only ascomycetous fungi able to produce AFP [89].

**Figure 1:**
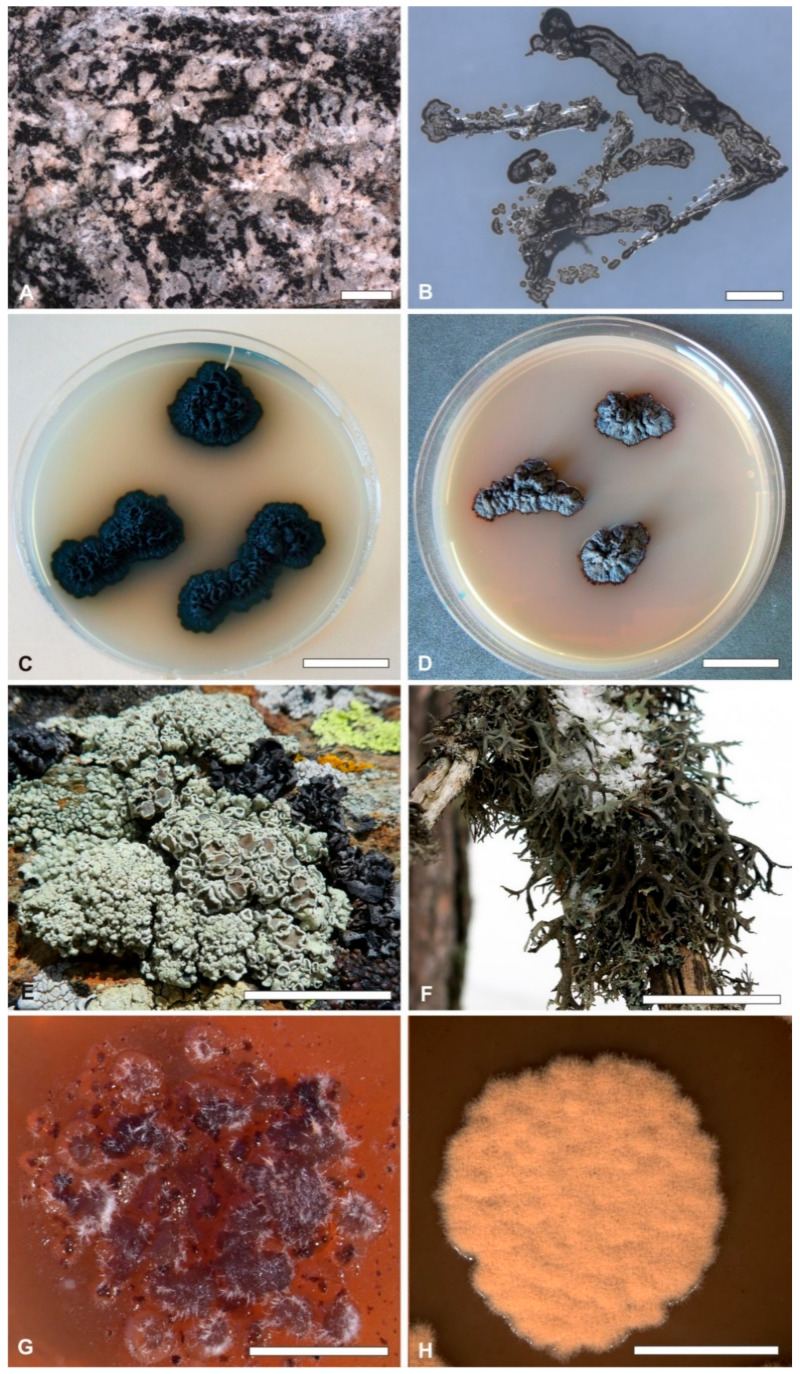
Fungi used in genomic studies. (**A**)Microcolonial black fungus *Lichenothelia calcarea* (L1799) [94], axenic culture of (**B**) the polyextremotolerant halophitic black yeast *Hortaea werneckii* EXF-6566 [91], (**C**) Knufia chersonesos (MA5789) and (**D**) *Cryomyces antarcticus* (MA5682); the lichen species (**E**) *Rhizoplaca melanophthalma* [134] and (**F**) *Pseudevernia furfuracea* [115]; axenic culture isolate of the lichen forming fungi (mycobiont) (**G**) *Arthonia radiata* [Appendix A] and (**H**) *Rhizoplaca melanophthalma* [136]. Photos (**A**,**B**,**E**,**G**,**H**) by Lucia Muggia, (**C**) by Donatella Tesei, (**D**) by Christian Voitl, (**F**) by Heinrich Meier. Scale bars: (**A**) 0.5 mm, (**B**) 1 mm, (**C**–**E**) 2 cm, (**F**) 5 cm, (**G**,**E**) 5 mm, (**H**) 1 cm.

*Microcolonial, rock-inhabiting, black fungi and black yeast*—Black, microcolonial (MCF), rock-inhabiting (RIF) fungi and black yeasts (BY) constitute a group of polyphyletic taxa, the majority of which have evolved in multiple orders within Ascomycota in the classes Dothideomycetes and Eutotiomycetes. These fungi are highly resistant to stressful environmental conditions and have adapted to harsh environments where life is brought to its extremes (Figure 1A–D). They share evolutionary traits, such as lack of teleomorphs and sexual reproduction, morula or clump-like growth, highly melanized cell wall, resistance to high UV radiation, radioactivity and desiccation, enormous heat and acid tolerance, slow growth rate, the ability to grow at high salt concentrations and oligotrophic conditions, to develop filamentous (hyphae) as well as yeast forms, to degrade aromatic compounds, and to develop pathogenicity. All these characteristics let define them polyextremotolerant, extremophilic organisms [48,50]. However, their genetic characterization has been so far hampered by difficulties in isolating and growing them in culture (due to their very slow growth rates or the requirement of peculiar conditions) and extracting their genomic DNA (due to the highly melanized cell walls). Nevertheless, genome sequencing combined with transcriptomics and proteomics studies (see further below in this contribution) are of key importance to explore gene content and genomic patterns that could be attributed to the multiple specializations evolved by these fungi. So far only a few, among all the known extremophilic species of black fungi and BY, could be sequenced. Full or partial annotations are available for the highly extremophilic *Cryomyces antarcticus* (CCFEE 534, MA 5682) [90], the halotolerant *Hortaea werneckii* (EXF-2000) [91], *Aureobasidium pullulans* (EXF-150, CBS100280) [92], *Friedmannomyces endolithicus* (CCFEE 5311), *Friedmanniomyces simplex* (CCFEE 5184), *Baudoinia panamericana* (UAMH 10762), *Acidomyces acidophilus* (BFW), *Hortaea thailandica* (CCFEE 6315). Draft genomes were presented for *Rachicladosporium antarcticum* (CCFEE 5527) and *Rachicladosporium* sp. (CCFEE 5018) [93], *Lichenothelia convexa* (LMCC0061, MUT5682), *Lichenothelia intermixta* (LMCC0543), *Saxomyces alpinus* (CCFEE5470, CBS135222), and *Saxomyces americanus* (LMCC0060, MUT5853) [94] and *Cladophialophora immunda* (CBS 110551) [95], *Exophiala mesophila* (CCFEE 6314) [96], and *Knufia petricola* (wild type MA5798) and its spontaneous non melanized mutant (MA5790) [97].

Genome analyses of *Cryomyces antarcticus* indicated that it experienced neither genome duplication nor a significant reduction of the genome in the course of adaptation to hostile conditions in the Antarctic [91]. Interestingly, the GC content of this fungus is in the middle range of the fungi, thus not reflecting the cryophilic ecology of the species. Although CG content is generally assumed to be related to the environmental temperature of an organism—with high CG content resulting in better stability under elevated temperature (as reported for thermophilic fungi, see above) and lower CG contents indicating an adaptation to cold environments—this was not recovered in the *C. antarcticus* genome. Also, the percentage of repetitive sequences in *C. antarcticus* was found to be 33%, i.e., the same as in the black yeast *E. dermatitidis*, but much higher than *Cladosporium sphaerospermum*, *H. werneckii* and *C. apollinis*, which have a 3–6% lower amount of repetitive sequences. The two gene families responsible for the melanin biosynthesis, i.e., laccase and PKS genes, are present in *C. antarcticus* genome with a lower number of genes than in other comparative species. It was speculated that this may be due to a lower rate of genetic diversification and by the fact that melanin production is essential, so that conservation of the involved genes would be a survival guarantee for this fungus [90].

The genome of the extreme halotolerant yeast *Hortaea werneckii* was sequenced to investigate the exceptional adaptability of this species to osmotically stressful conditions [91] (Figure 1B). *H. werneckii* genomes resulted in 51.6 Mb, much larger than most phylogenetically related fungi, having almost twice the usual number of predicted genes (23333_) [93]. The large genetic redundancy and the expansion of genes encoding metal cation transporters emerged as the most striking characteristics. Furthermore, although no sexual state of *H. werneckii* has been described yet, a mating locus with characteristics of heterothallic fungi was also found. A revision of *H. werneckii* genome, performed on data obtained by long read and single-molecule sequencing, confirmed a relatively recent whole genome duplication (WGD; [98]), as two highly identical gene copies of almost every protein were identified. The analysis of predicted metal cation transporters showed that most types of transporters experienced several gene duplications and are present in a much higher number than expected [92,99]. These genomic features were found to be consistent with previous studies reporting on the increases in genomic DNA content triggered by exposure to salt stress [93]. In the firstly obtained genome sequence of *H. werneckii* [91] Gostincar et al. [99] found that the fungus has a diploid genome, consisting of two sub-genomes with a high level of heterozygosity, which is in contrast to the majority of the fungi that are haploid. To explain the origin of this diploid genome in *H. werneckii* Gostincar et al. [99] sequenced and compared the genomes of eleven strains isolated from different habitats and geographic locations. Of these, nine were diploid and two haploid strains and showed that the reference genome of *H. werneckii* (EXF-2000) was likely formed by hybridization between two haploids and not by endoreduplication [99], as suggested originally by Lenassi et al. [91]. The new analyses showed that the genomes and predicted proteomes contained more than 97% of identifiable fungal Benchmarking Universal Single-Copy Ortholog genes/proteins; these were single copy in two genomes (the haploid strains) while in the other genomes more than 60% were duplicated (the diploid strains; [99]).

*Aureobasidium pullulans* is another extremotolerant black yeast-like micromycetes exploited for the production of the polysaccharide pullulan and the antimycotic aureobasidin A. Beside thriving as extremophile, *A. pullulans* also causes opportunistic human infections and is used as a biocontrol agent in agriculture. *De-novo* genome sequencing was performed to mine its genome and link the fungus life-style with its genomic traits [92]. Four varieties of *A. pullulans* were sequenced and the results showed that the genomes encoded most of the enzyme families involved in degradation of plant material and many sugar transporters, and have genes possibly associated with degradation of plastic and aromatic compounds [92]. Predicted proteins were those believed to be involved in the synthesis of pullulan and siderophoresbut not of aureobasidin A, in stress-tolerance (such as aquaporins and aquaglyceroporins), transportation of alkali-metal cations, synthesis of compatible solutes and melanin, and of the components of the high-osmolarity glycerol pathway. The features of the four genomes were further used to support differentiation between the varieties and served to re-define them as four separate species (i.e., *A. pullulans*, *A. melanogenum*, *A. subglaciale* and *A. namibiae*; [92]).

*Friedmanniomyces* is a genus of exclusively endolithic fungi endemic of the Antarctic Continent [100]; to date it includes the two species of *F. endolithicus* and *F. simplex*. In particular, *F. endolithicus* occurs as the most commonly isolated species in endolithic communities of the ice-free areas from the McMurdo Dry Valleys of the Victoria Land (Antarctica), one of the coldest and most hyper-arid deserts on Earth. The genome of *F. endolithicus* was compared to phylogenetically closely related BY and black fungi (*F. simplex*, *B. panamericana, A. acidophilus*, *H. thailandica* and *H. werneckii*) and was found to be as large as that of *H. werneckii*, i.e., much larger than genomes of most other black fungi so far sequenced [101]. *F. endolithicus* genome was enriched with some characters that confers stress and cold tolerance [101]; in particular, a few gene ontology (GO) terms related to meristematic growth and cold adaptation were found to be unique for it.

Many fungi belonging to the genera *Exophiala* and *Cladophialophora* (Chaetothyriales), and *Pseudallescheria* (Microascales) grow in polluted environments and metabolize hydrocarbons as the sole source of carbon and energy. *Cladophialophora immunda* was characterized for its ability to degrade up to 65% of the toluene supplied [102,103] and was chosen for a genomic and transcriptomic study by Blasi et al. [95]. The analyzed strain was sampled from environmental polluted sources, but the species is also an opportunistic human pathogen, causing subcutanoeus phaeohyphomycosis [104]. The dual ecology was hypothesized to have arisen from the hydrocarbon degrading pathways, which would allow the fungus to use the neurotransmitters for their own energy metabolism [103]. In *Cladophialophora immunda* five gene clusters coding for toluene-degrading pathway were identified. The comparison of fungal and bacterial toluene-degradation pathways let identify 8 genes responsible for toluene degradation steps shared by *Cladophialophora immunda* and *Pseudomonas* spp. Four of these genes were suggested to have been horizontally transferred from bacteria [95]. The genomic analysis of significantly enriched protein domains also led to interesting observations about the ecology of *C. immunda*. For example, proteins involved in complex carbohydrates degradation and sugar transport are overrepresented and might suggest that the fungus evolved in an oligotrophic and polluted environment [95].

At last but not least, ant-associated black yeasts represent a further interesting group of extremophilic fungi. These fungi belong to the order Chaetothyriales and have specialized symbiotic associations with ants; they present evolutionary adaptations that allow them to assimilation and tolerate the toxic compounds produced by the ants. Ant-associated Chaetothyriales can be classified into three ecological categories based on the nature of the fungal-ant interaction: living in ant-occupied domatia in plants, colonizing ant-made carton structures, and living as parts of fungus-gardens of leaf-cutter ants [105]. The genome of the first ant-associated black yeast was sequenced for *Phialophora attae*, isolated from the cuticle of tropical ant gynes [106]. More recently whole genome of selected domatia-inhabiting members of the order Chaetothyriales were sequenced [107] and compared with 24 previously sequenced black yeasts of the chaetothyrialean families Herpotrichiellaceae, Cyphellophoraceae, and Trichomeriaceae presenting different lifestyles. Moreno et al. [107] found that these genomes are rather compact and analyzed key gene families (such as cytochrome P450, transporters, alcohol dehydrogenase, CAZymes) thought to be essential for the success of these fungi in colonizing ant nests as well as extreme and toxic habitats, and which might also play a role in recurrent opportunism in the family Herpotrichiellaceae. By comparing the content of protein functional domains identified in the Chaetothyriales colonizing ant domatia against the family Herpotrichiellaceae, the authors verified 23 significant gene family contractions. These contractions provide specific genomic signatures of black domatia-associated fungi, being unique among the Chaetothyriales. Further comparative analyses with distantly related ascomycetes showed that highly conserved homologs of pectate lyase are shared with some important fungal plant pathogens, while other polysaccharide lyase families have been lost completely in black yeasts associated to ant domatia. Alternatively, enzymes linked to degradation of cellulose, such as the glycoside hydrolase subfamilies were completely absent in the domatia-associated species. Instead, the domatia-associated fungi harbor the highest number of biosynthetic polyketide synthase (PKS) clusters predicted in Chaetothyriales. 19 biosynthetic clusters were identified for type I PKS (t1pks), being the most prevalent cluster type ranging from 10 to 14 copies in different strains. Type III PKS cluster (t3pks), was not observed in the domatia-associated species although it was previously reported in Herpotrichiellaceae, Cyphellophoraceae, and Trichomeriaceae. Interestingly, the genomes of four ant domatia-associated strains contained repetitive elements in an amount inversely proportional to the genome size of these species, which have the smallest genomes reported in Chaetothyriales so far [49]. Also, the composition of CAZymes found in the black yeasts associated to ant domatia suggests that these organisms are not efficient polysaccharide decomposers [108]; this discovery contrasts with the classic view of mutualistic fungus-ant relationship, where the fungal partner degrades plant material incorporated by the ants [109].

### 2.5. Lichenized Fungi

Lichens are stable, mutualistic, symbiotic associations between specialized fungi (the mycobionts) and populations of unicellular green algae (the phycobionts) or cyanobacteria (the cyanobionts; [110,111]). The majority of the ascomycetous lichenized fungi are comprised in the class Lecanoromycetes ([111] while only a few species belong to Dothideomycetes and Eurotiomycetes and represent important evolutionary links between the free living, the parasitic and the lichenized fungal life styles. Though lichens represent key organisms in many ecosystems, they have been still poorly investigated by means of -omics approaches. This is mainly due to (i) the complexity of the lichen thalli—beside the two major symbionts, lichens host also additional fungi (yeast and filamentous fungi as parasites or cryptic endolichenic) algae and bacteria [112,113,114], (ii) the impossibility (still) to reproduce and maintain in culture a whole lichen symbiosis, and (iii) the particularly challenging isolation of the lichen mycobionts, which requires lots of attempts and it can take up long time (from several months up to one year) before obtaining enough fungal mycelium to be used for experiments and/or sequencing. Nevertheless, studies demonstrated that it is possible to gain relatively complete genome data for lichen mycobionts bypassing aposymbiontic isolation in culture [115,116]. A list of all reviewed genomic works concerning licghen symbioses is shown in Appendix A.

*Genome sequencing—*23 lichen mycobiont genomes—of the about the 20,000 known lichen species [117]—have been sequenced and are publicly available (6 July 2020, NCBI), but others have been sequenced, assembled and partially annotated, starting from metagenome sequencing of entire thalli or from culture isolates [118,119,120,121,122,123]. Two lichen transcriptomes of the species *Cladonia rangiferina* and *Cladonia grayi* [123,124] and some mitochondrial genomes of *Cladonia* spp. [125], *Peltigera* [126] and *Usnea* mycobiont species [127] were also published. All together these genomes have been analyzed to answer questions about the evolution of lichen symbiosis [119,120,121,122,123,128,129,130,131], homothallism in lichen-forming fungi [132], or to study horizontal gene transfer and hybridization processes [118,133]. Additional studies used sequenced nuclear and/or mitochondrial mycobiont genomes to assemble comprehensive datasets for phylogenomic or population genomic analyses in closely related taxa [133,134,135,136]. Though representing still a limited number of genomes, all these studies already provided structured data and genomic resources for further, more extensive phylogenomic analyses and investigations aiming to understand the basis of adaptation, niche differentiation and population genomics (re-sequencing natural population), and to study the impact of different reproductive strategies on the evolution of lichens.

So far four genomic studies were carried out on five in vitro isolated mycobionts, i.e., *Cladonia grayi* [123], *Endocarpon pusillum* [130], *Lasallia hispanica* [131], *Evernia prunastri* and *Pseudevernia furfuracea* [132] (Figure 1F). The first two also considered the corresponding isolated photobionts, i.e., *Asterochloris glomerata* and *Diplosphaera chodatii*, respectively, and performed co-culturing of the two symbionts to complement genome analyses with transcriptomic data [123,131]. These studies provided the annotation of several genes codifying for secondary metabolites biosynthesis, mating type loci, signal peptide domains essential for the symbiotic recognition in the lichen, genes influencing the symbionts’ environmental stress resistance and genes relevant for the regulation of nutrient interactions, such as transporters involved in carbon and nitrogen exchanges [123,130,131]). For most of the symbiotically relevant genes identified in *C. grayi*, their homologs were recognized in non-lichen fungi and algae, likely representing variants modified by the symbiotic lifestyle. This suggested that lichens evolved mainly through the accumulation of scattered regulatory and structural changes in already available genes rather than through key innovations [123]. This evolutionary scenario proposed by Armaleo and coworkers [123] supports the hypothesis that there were multiple pathways for fungi, algae and cyanobacteria to evolve into lichens, which is indeed consistent with the few independent evolutionary origins of lichenized fungi [137].

The evolution, including gains, losses and horizontal transfer events, of genes codifying for ammonium transporters (MEP α) involved in nitrogen exchanges was in detail investigated by whole genome sequencing of eight lichenized fungi representatives of key lineages in Pezizomycotina [118]. Interestingly, the MEP α genes have been lost in the majority of the non-lichenized and in some lichenized lineages while retained in few others, suggesting that they may not be intimately involved in the lichen symbiosis. The retention/loss of the MEP α genes in the genomes of certain mycobionts and the production of the actively synthesized enzyme does not seem to be mandatory to supplying nitrogen to the lichenizing fungus and does not correlate with the nitrogen availability in the environment. However, the authors speculated that the MEP α genes still might be involved specifically in balancing the nitrogen budget between the fungal and the green algal symbionts [118].

Whole genome sequencing of lichen mycobionts proved successful also to characterize the allelic variants of the MAT loci [132]. In lichens, homothallism (self-fertility) was hypothesized to be the predominant reproduction strategy of the mycobiont. However, the analyses conducted on 41 genomes of lichen-forming fungi representing a wide range of growth forms and reproductive strategies in the class Lecanoromycetes, revealed the complete lack of genetic homothallism and suggested that lichens evolved from a heterothallic ancestor. This was argued to be related to the symbiotic lifestyle of the fungi and to represent a key innovation that has contributed to the accelerated diversification rates of the lichen mycobionts [132].

Wilken et al. [129] presented very recently the genome sequence of the mycobiont *Physcia stellaris*, a nitrophytic lichen species which outcompete native lichen assemblages in polluted environment and is used as bioindicator. Its genome will now be a reference to facilitate future studies in understanding the genetic mechanisms behind the adaptation of lichenized fungi to high nitrogen environments. This genome is an improved hybrid assembly which was sequenced and assembled from a combination of long-read Nanopore and short-read Illumina data. It contains 74 regions that had genes associated with secondary metabolite biosynthesis, in particular comprised of 20 type I and a single type III PKSs, 15 non-ribosomal peptide synthetases (NRPSs), and 16 NRPS-like fragments [129].

*Metagenome skimming and RADseq—*In the past few years lichens have been successfully used also as suitable systems to demonstrate the utility and the efficiency of different techniques for generating genomic data, such as whole genome shotgun (WGS) sequencing/metagenome skimming (i.e., low coverage shotgun sequencing of multi-species assemblages and subsequent reconstruction of individual genomes; [138,139,140]) and restriction site-associated DNA sequencing (RADseq; [135,136]). As lichens represent consortia of many diverse microorganisms with small genomes, they are particularly intriguing because of their wide distribution, adaptive strategies and the potential mycobiont-photobiont coevolution. Therefore, they can serve very well as microecosystem to improving estimates of microbial biodiversity for the purpose of understanding ecological communities and their evolutionary processes [138].

Meiser et al. [115] applied metagenome skimming to assemble the genomes of the two lichens *Evernia prunastri* and *Pseudevernia furfuracea* based on sequences derived from whole lichen thalli. Subsequently, the authors assessed quality and completeness of the metagenome-based assemblies using as reference the genome assemblies obtained from the pure cultured strains of the two corresponding mycobiont species. In doing so the authors were able to reconstruct fungal genomes from uncultured lichen thalli covering most of the gene space (86–90%; [115]). Metagenome skimming was thus suggested to be a promising technique to facilitate genome mining of lichen-forming fungi by circumventing the time-consuming, sometimes unfeasible, step of aposymbiotic cultivation. Up to 2019 only two publicly available lichen genomes have been assembled from metagenomic data (i.e., that of *Cetradonia linearis* and *Alectoria sarmentosa*, [116,141]), and these are far from being complete, still indicating how challenging is genomeassembling from metagenomic data. However, just recently Greshake Tzovaras et al. [140] have successfully reconstructed the hologenome of the mycobiont *Umbilicaria postulata* combining short Illumina with long PacBio reads with a comprehensive assembly scheme. Though no genome assembly procedure has ever being standardized, the authors took care to target different components of the holo-genome with different assembly methodologies, to include taxonomic assignments on the contig level, to perform a merging of contigs from different assembly approaches that were assigned to the same taxonomic group, and to finally perform a scaffolding step [140].

A new exploring avenue has implemented WGS to detect hidden species diversity and demonstrated its better performance against amplicon-based methods (eDNA metabarcoding; [139]). Here, WGS detected even up to four times more species than the amplicon-based method and was thus suggested to be considered as a further tool for molecular taxonomists in pursuit of conservation and restoration efforts, as it facilitates a more general understanding of species distributions and community structure [139]. Notwithstanding that both WGS and eDNA metabarcoding rely on low abundance DNA being extracted in sufficient quality and quantity to be detected in downstream analysis, Keepers et al. [139] reasonably highlighted that WGS performs better as it overcomes those difficulties encountered in metabarcoding when using universal primers for library construction.

Genome scale data obtained by RADseq were instead implemented in fine scale population genomic and phylogenomic analyses of closely related lichen species of *Rhizoplaca* (Figure 1E,H)—characterized by a well-studied diversification history [135,136]. The aims of the study were to trace population dynamics and to better resolve previously phylogenetic relationships based on multiple loci [135,136]. RADseq proved effective to generate symbiont-specific phylogenomic data from metagenomic reads, as it lets select thousands of homologous loci but at the same time it reduces their number across the whole genome. In this case phylogenetic reconstructions using RADseq loci recovered diversification histories consistent with a previous study based on more comprehensive genome sampling [134]. Furthermore, RADseq loci were found to resolve relationships among closely related species, which were otherwise indistinguishable using a phylogenetic species recognition criterion [135,136].

*Mitochondrial genome analysis in lichens—*Studies reporting on mitochondrial genomes in lichens are only a few. These have highlighted the high dynamic evolution of mitochondrial genomes and suggested that recent, broad-scale genome streamlining via loss of protein-coding genes as well as noncoding, parasitic DNA elements are ongoing processes in lichens. Pogoda et al. [142] examined patterns of mitochondrial genome evolution specifically looking at presence/absence of introns, intron size, number and position in 58 species (21 genera) of lichenized ascomycetes from diverse ecological niches. Genome size proliferation was evidenced by intron gain, while genome streamlining was due to loss of mitochondrial introns over a short evolutionary timescale [142]. Substantial variation of mitochondrial genomes was detected even between sister species, in a genus where species differed by fivefold in intron number. Brigham et al. [125] instead analyzed mitochondrial genomes of 11 *Cladonia* species, including nine new mitochondrial genome assemblies and annotations. The 11 mitochondrial genomes varied in size, intron content, and complement of tRNAs while 15 protein-coding genes were newly annotated. Among these the authors found the atp9 gene coding for an important energy transporter that has been evolutionarily lost in some mycobiont mitochondria. These 11 genomes were also used to select five loci (nad2, nad4, cox1, cox2, and cox3) for a concatenated alignment to infer a Bayesian phylogenetic relationship among species. This phylogenetic inference is consistent with previously published phylogenies and strengthens the utility of the new regions in reconstructing phylogenetic histories [125].

### 2.6. Phylogenomics and Population Genomics

*Phylogenomic studies—*The earliest phylogenomic studies focused on the backbone of the fungal tree of life or on higher taxonomic level relationships, which were difficult to be resolved using single or multi locus phylogenies [5,143]. Liu et al. [144] were among the first in taking advantage of new data provided by both nuclear and mitochondrial genome projects for selected species of Taphrinomycotina (Ascomycota) and compared two large datasets, the first with 113 nuclear and the second with 13 mitochondrial protein sequences. Their study supported the monophyly of Taphrinomycotina and its further divergence as a sister group to Saccharomycotina and Pezizomycotina. Ebersberger et al. [18] used 99 fungal genomes and 109 fungal expressed sequenced tag (EST) sets to reconstruct a kingdom-wide fungal phylogeny, including the early branching lineages of Ascomycota and Basidiomycota. Later, Liu et al. [145] performed a more detailed comparative genomics analysis of genome sequences including 55 ascomycetes and 26 basidiomycetes species and detected 81 universal markers, 875 homologous genes and a conserved contig in the glucose-regulated protein gene. The authors recovered ascomycetes and basidiomycetes forming distinct clusters, with each set of taxa having a high coefficient of relatedness and forming distinct groups in a phylogenetic tree based on a conserved contig in the glucose-regulated protein gene. These results provided evidence that basidiomycetes may be derived from ascomycetes but are genetically differentiated at the genomic level [145].

An alternative genomic approach has been overtaken by Choi and Kim [17] who chose to reconstruct the fungal tree of life using a “genome tree” starting from whole proteome sequences to estimate similarity between two organisms without implementing a multiple sequence alignment (MSA). It emerged that there are only three earliest diverging and deepest branching major fungal groups in the proteome tree, i.e., Monokarya, Basidiomycota and Ascomycota [17]. Phylogenomic studies focusing on Ascomycota are in general still limited and mainly centered on some, usually industrially or medically interesting lineages, such as *Saccaromyces cervisiae*, or animal and plant pathogens, while other groups received less attention, or even still lack genome data at all.

**Figure 2:**
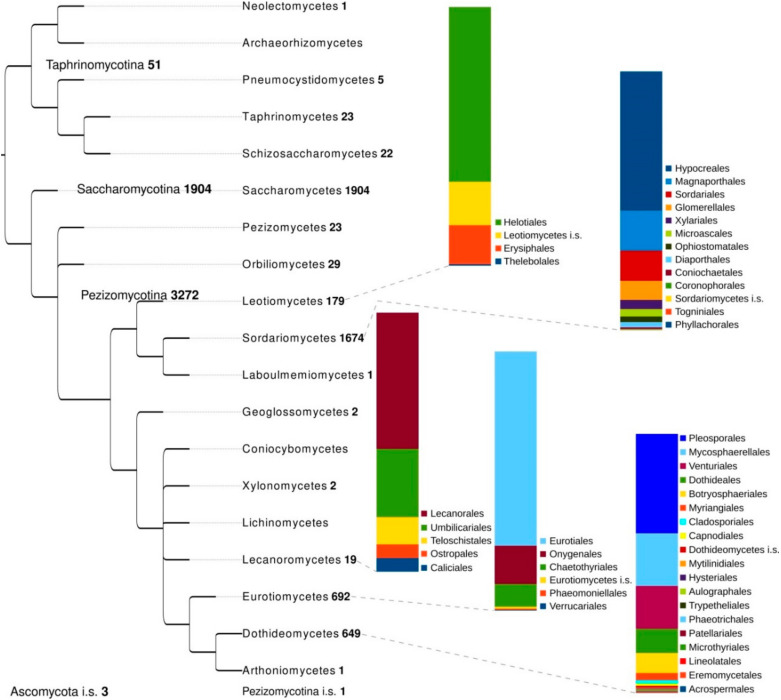
Ascomycota tree of life. Topology, sub-phyla and classes included are taken from Spatafora et al. (2017). Next to each taxa is reported the number of assemblies retrieved from NCBI database (6 July 2020). For the five most diverse classes in Ascomycota also orders that have at least one genome sequenced are reported; bar charts are in percentage. Data were retrieved from NCBI assembly reports, using the ncbi-genome-download script (https://github.com/kblin/ncbi-genome-download/) [148] and a custom script which exploits NCBI Entrez Programming Utilities to retrieve taxonomy.

The classes Dothideomycetes, Eurotiomycetes and Leotiomycetes were so far the subjects of large scale phylogenomic studies in ascomycetes (Figure 2). These classes include a huge diversity of fungi, many of which are polyextremotolerant, melanized taxa having different ecologies and versatile life styles which evolved multiple times. Major representatives of these classes are plant pathogens with broad host spectra, etiologic agents of several diseases in vertebrate hosts, rock inhabiting fungi, lichen parasites and lichenized fungi (see above). Analyses have mainly chosen to consider the evolution of certain life styles individually and searched for genomic feature potentially involved in the expression of those phenotypes. Ohm et al. [146] compared genome features of 18 members of Dothideomycetes, including 6 necrotrophs, 9 (hemi)biotrophs and 3 saprotrophs, to analyze genome structure, evolution, and the diverse strategies of pathogenesis. It emerged that Dothideomycetes most likely evolved from a common ancestor more than 280 million years ago; though their genome sequences differ dramatically in size due to variation in repetitive content, they show much less variation in number of (core) genes. In the two major dothideomycetes order of plant pathogens, Capnodiales and Pleosporales, genes related to pathogenesis were found to be more abundant in Pleosporales than in Capnodiales. Interestingly, many of these genes were enriched in proximity to transposable elements, thus suggesting a faster evolution because of the effects of repeat induced point (RIP) mutations [146].

The class Dothideomycetes was very recently also the focus of an alternative genomic approach implemented by Haridas et al. [147] and based on machine-learning methods, in which the authors presented the largest genome comparison, introducing 55 newly sequenced dothideomycetes genomes. A total of 101 genomes of dothideomycetes were used to assemble a dataset of 738 single-copy orthologs. The high-confidence phylogeny reclassified 25 species, providing a clearer picture of the relationships among the various families, and indicated that pathogenicity evolved multiple times within the class. A subset of known pathogenic and saprobe species were selected to train the machine-learning methods to identify the most informative features for differentiating between saprobes and pathogens. In doing this it was possible to identify several gene family expansions and contractions which are congruent with changes in the lifestyle across the whole class [147].

The most inconspicuous fungal species in Dothideomycetes, such as those representing rock-inhabiting fungi (RIF), instead, were neglected by genomic researches due to the difficulty to retrieve them in nature, isolate and grow them axenically in vitro. Only recently a few RIF genomes became available [94,95,97,101], though they have not been fully annotated yet. These were considered by Ametrano et al. [94], who assembled different phylogenomic datasets including four newly sequenced species of the two RIF genera *Lichenothelia* and *Saxomyces* and 238 previously available genomes. The influence of tree inference methods, supermatrix vs. coalescent-based species tree, and the impact of varying amounts of genomic data was explored by to better resolve relationships in Dothideomycetes [94]. This phylogenomic study, beside supporting the rock-inhabiting lifestyle as ancestral of the whole class, showed that if samples with a high amount of missing data (up to 90%) were included, a subset corresponding to about 20% of the whole supermatrix would be enough to provide a phylogenetic accuracy which does not improve significantly when the amount of data is further increased [94].

Coleine et al. [101] used 24 genomes of black fungi belonging to Dothideomycetes and the sordariomycetes *Neurospora crassa* and *Sordaria macrospora* as outgroups, to infer the phylogenomic relationship of the Antarctic strains of *Friedmanniomyces endolithicus*, *F. simplex* and *Hortaea thailandica*. The whole genome phylogeny was consistent with previous analysis based on the sequence of the ITS-SSU regions, supporting indeed that *F. endolithicus* and *F. simplex* are monophyletic in Dothideomycetes while the Antarctic strain *H. thailandica* is sister to the halotolerant black fungus *H. werneckii* EXF-2000.

A special attention to the class Eurotiomycetes was given by Teixeira et al. [49] who performed a large scale phylogenomic analysis on a set of 23 newly sequenced genomes of the main human opportunistic fungi within the order Chaetothyriales (see above). The phylogenomic position of Chaetothyriales was inferred based on 264 single-copy orthologous protein clusters identified among 53 fungal species included in the whole dataset [49]. The genome scale inference revealed *Coniosporium apollinis* more closely related to the order Botryosphaeriales in Dothideomycetes than to Chaetothyriales, similarly as Ruibal et al. [149] previously suggested in a three gene phylogeny. Chaetothyriales turned to be closely related to Verrucariales and Phaeomoniellales, and the early and major black yeast lineages of Herpotrichiellaceae and Cyphellophoraceae were estimated to be contemporaneous and emerged around 50–75 MYA during/after the Cretaceous-Paleogene (K-Pg) extinction event, underling that species radiation of Chaetothyriales took place in the class more recently than that of Onygenales [49].

The fungal family Aspergillaceae (Eurotiomycetes) contains about 1000 known species, mostly in the genera *Aspergillus* and *Penicillium*, of which several are used in food, biotechnology and drug industries, while others are dangerous human and plant pathogens. Steenwyk et al. [150] provided a robust and comprehensive evolutionary genomic roadmap for this important lineage, which is expected to facilitate the examination of the diverse technologically and medically relevant traits of these fungi in an evolutionary context. The authors indeed used 81 genomes spanning the diversity of *Aspergillus* and *Penicillium* to construct a 1668-gene data matrix to infer a robust phylogeny and identify poorly resolved branches. This huge data matrix was elaborated with three different maximum likelihood schemes for tree reconstruction and using both site-homogenous and site-heterogeneous models. The phylogenomic results indicated whether topological incongruence was generated by incomplete lineage sorting, hidden paralogy, hybridization or introgression, and reconstruction artifacts associated with poor taxon sampling [150]. In general, this work aimed at providing a reference template for phylogenomic identification of resolved and controverted branches in densely genome-sequenced lineages across the tree of life [150].

Genomic data available from specimens representing both sexual and asexual morphs from across the genetic breadth of the class Leotiomycetes were gathered by Johnston et al. [151] who took care to include generic type species. The authors released 10 newly sequenced genomes and presented a phylogeny based on up to 15 concatenated genes across 279 specimens including genes extracted from 72 of the genomes available for the entire class. A larger scale phylogeny based on 3156 genes from 51 selected genomes was additionally performed to test the statistical support for the deepest branches in the phylogeny. The phylogenomic analyses based on the 15 concatenated genes helped to resolve many family-level relationships, while a new order and two new families were introduced, along with the validation of an existing family name [151]. In this work the authors stressed the importance of the phylogenomic approach to offering a framework for enabling future taxonomically targeted studies using pondered specimen selection.

At last but not least, phylogenomic analyses of entomopathogenic fungi were performed on the most studied genera of *Beauveria, Cordyceps* and *Metarhizium* and suggested that their ancestors were plant endophytes or plant pathogens and that entomopathogenicity has evolved as an acquired trait [69,70,71]. These inferences also showed that the species *Beauveria bassiana* is polyphyletic and closely related to *Cordyceps militaris*, and it evolved into insect pathogens independently of the *Metarhizium* lineage [69,70,71].

*Population genomics—*Population genomic analyses can provide insight into the evolutionary history of populations by genome-wide measures of genetic variation, including gene flow, population size and reproductive system, as well as identifying genomic regions experiencing recombination or selection [152]. Fungal population genomics encompasses detailed genetic analyses of natural populations, comparative genomic analyses of closely related species, identification of genes under selection, and linkage analyses involving association studies in natural populations or segregating populations resulting from crosses [153]. However, population genomic studies are still rare, particularly because they require large genomic dataset from numerous individuals of the same, or closely related species. Nevertheless, they are becoming key to discover genetic mechanisms underlying phenotypes associated with adaptive traits, such as pathogenicity, virulence, fungicide resistance, and host specialization [153].

A very comprehensive population genomic study has been recently completed on the entomopathogenic fungus *Beauveria bassiana*. Mei at el. [154] reported on the ecological and evolutionary scenario of the *Beauveria* population genomics over 20 years after releasing exotic strains to control pine caterpillar pests. They found that the population was largely clonal in nature and the released strains could persist in the field for a long time (20 years) but with low recovery rates. The authors also showed how *Beauveria* population evolved under balancing selection even with substantial population replacements once a decade time through the joint effects of adaptation, non-random outcrossing and isolate migrations. With this study Mei at el. [154] paved the way for researches on population evolution of other species of entomopathogens, especially for biocontrol species with narrow host ranges.

*Fusarium graminearum* is the primary cause of *Fusarium* head blight (FHB) in cereals and represent a significant threat to food safety and crop production. Kelly and Ward [155] sequenced the genomes of 60 diverse *F. graminearum* isolates to elucidate population structure and identify genomic targets of selection within major populations in North America. The authors also assembled the first pan-genome for *F. graminearum* to clarify population-level differences in gene content which could contribute to pathogen diversity. Their analyses highlighted 121 genes with population-specific patterns of conservation, while high diversity, frequently recombining regions -containing genes potentially involved in pathogen specialization, were characterized by distinct signatures of selection for genomic divergence. Those genes that differentiated populations had predicted functions related to pathogenesis, secondary metabolism and antagonistic interactions, though a subset had unique roles in temperature and light sensitivity. Their results indicated that *F. graminearum* populations are distinguished by dozens of genes with signatures of selection and an array of dispensable accessory genes, suggesting that this pathogenic fungus may be equipped with different traits to exploit the agroecosystem [155]. Within the *F. graminearum* species complex (FGSC), *Fusarium asiaticum* is one member which causes sever yield losses of wheat, barley and rice in Asia. Yang et al. [156] conducted a population genomic studies on the mitochondrial genomes of 210 strains of *F. asiaticum* isolated from wheat or rice from several geographic areas in seven provinces in Southern China. The sequenced, assembled, and annotated mitogenomes proved to be extremely conserved and variation was mainly caused by absence/presence of introns harboring homing endonuclease genes. The authors suggested that these variations found could be utilized to develop molecular markers for track and trace of migrations within and between populations. Also, a SNP analysis demonstrate the occurrence of mitochondrial recombination in *F. asiaticum*, as it was previously found for *F. oxysporum*.

Another example of population genomics comes from the study performed on *Verticillium dahliae*, a plant pathogen which causes vascular wilt on a variety of different hosts, but induce defoliation only in the three hosts cotton, olive and okra [157]. Previous genomic researches have provided evidence for cryptic sexual reproduction among clonal lineages and investigated molecular mechanisms that foster pathogenicity and virulence of this fungus ([157] and references therein). These studies also reveal that mechanisms of host adaptation in *V. dahliae* may evolve through chromosomal rearrangement or horizontal gene transfer from other pathogens, such as *Fusarium oxysporum*. However, the mechanistic bases for the defoliation phenotype (D) of *V. dahliae* were only recently uncovered by Zhang et al. [157] who performed whole-genome re-sequencing and demonstrated that the defoliation and high virulence of D pathotype is the result of secondary metabolites, the biosynthesis of which is controlled by genes in the lineage-specific region G-LSR2 in *V. dahliae*.

## 3. Progress and Applications of Sequencing Technologies to Fungal Genomes

### 3.1. Genome Sequencing

*Early history of genome sequencing in Ascomycota—*Since the first whole genome sequencing of the fungus (yeast) *Saccharomyces cerevisiae* was completed [158,159], two main technological revolution in sequencing technology took place. In 1996, the sequencing of the relatively small and compact yeast’s genome (about 12 Mb) required a huge international effort of about 600 scientists and several million dollars funding. The event was even more relevant as it was the first complete genome within the Eukarya domain, in accordance with the role of *S. cerevisiae* as a convenient model organism for eukaryotes and Fungi. In that case, and in the following years, during the sequencing projects of progressively larger eukaryotic genomes, a major challenge was the overall limitation of the sequencing technique (Sanger dideoxy), but also the construction of clone libraries in high-capacity vectors and the tedious filling-in of the gaps in clone coverage [159]. The race towards progressively larger and more complex genomes continued during the last years of the 20th century and the early 2000s, and other relevant fungal genomes, such as *Neurospora crassa*, were on the way to be completed in those years [160]. From a technical point of view, fungal genomes were considered the perfect target for a genome sequencing program, being relatively small and having high gene density and few repetitive regions. The next fungal genomes were completed much quicker than *S. cerevisiae*, but the project costs were still in the order of 10^5^–10^6^ $ for a small to medium fungal genome. In 2005 over 40 fungal genomes were available, for most of them, the reduction in cost and time was mainly determined by the use of whole genome shotgun sequencing approach coupled with new assembling approaches instead of the classical clone-by-clone approach. Contrary to the clone-by-clone approach, whole genome shotgun sequencing does not need a physical map of the genome, as it only relies on the overlap of the sequencing reads to assemble the entire genome. Either methods have their pros and cons, therefore, they were often used together to optimize the quality of the genome and its sequencing cost [161]. Whole genome shotgun sequencing approach rapidly improved and became the standard method, producing fungal genome sequences with high accuracy and long-range contiguity at ever-reduced cost [162]. The success of this strategy was determined by the reduced complexity of library preparation methods but, above all, by the advent of the high throughput sequencing (HTS) technologies, which represented a real revolution for genomics, dramatically increasing the pace of Ascomycota genome sequencing and therefore the availability of genomic data, with a 100-fold increase of sequenced fungal genomes in 15 years (5230 assemblies belonging to ~2000 species; 6 July 2020NCBI).

*Second generation sequencing technologies—*HTS, also known as next-generation sequencing (NGS), massively parallel sequencing, deep sequencing or 2nd generation sequencing, is a group of DNA sequencing technologies which use the principle of sequencing by synthesis. Though there are differences in library preparation, in the way sequencing is performed, and nucleotide incorporation is detected, these technologies have in common the following basic characteristics: shotgun sequencing of randomly fragmented genomic DNA (or cDNA) is performed without the need of cloning via a foreign host cell. Instead, adapter sequences and primers are ligated to the fragmented DNA for construction of template libraries. Library amplification is performed on a solid surface or on beads while isolated within miniature emulsion droplets or arrays. Nucleotide incorporation is monitored by luminescence detection (e.g., Solexa-Ilumina) or by changes in electrical charge (e.g., IonTorrent) during the sequencing procedure [163]. NGS generates many millions of nucleotide short reads in parallel (i.e., in the same sequencing reaction) during each run, therefore performing a much higher throughput than any Sanger sequencer. The higher sequencing error rate of these technologies, compared to Sanger sequencing, is compensated by the possibility of extremely high genome coverage. Though these technologies made available genomic data to a much wider number of research groups, the possibility to implement comparative genomics studies was still limited, because in many cases the resulting genome assemblies are drafts, with quality and contiguity far from those of a chromosome-level assembly. However, it is important to consider whether the genome needs to be sequenced to high accuracy, or whether a draft-level assembly is good enough to pursue a specific project aim. The limitations of whole genome shotgun sequencing using NGS are due to PCR biases, which reduce the complexity of the libraries and can lead to low coverage of high GC regions, but also to the difficulty in resolving repetitive regions of the genome using sequencing reads and fragmentsshorter than the repetitive region itself. The impact of GC and PCR biases in general, was mitigated using PCR-free library protocols [164]. Alternatively, the contiguity of the assemblies produced with short reads benefited from the use of longer insert size libraries coupled with paired reads technology [165]. This latter is one of the reasons that made Illumina the dominant second generation sequencing technology. Though these technologies enormously contributed to make genome sequencing easier, and four to five orders of magnitude cheaper than before, the development and the introduction of a new generation of sequencing technologies is rapidly changing the scenario again.

*Third generation sequencing technologies*—In 2009 two single-molecule sequencing (SMS) methods were published [165,166,167], one of them leading to the currently established Pacific Biosciences (PacBio) Single Molecule Real Time (SMRT) technology. Nanopore sequencing technology, instead, which was pursued for about thirty years [168] and improved in the last few years [168,169,170,171], became available for selected users in 2014. The distinguishing features of third generation sequencing (TGS) technologies are (i) the SMS, without the need of amplifying target DNA, and (ii) real-time sequencing, (iii) the ability to produce extremely long reads.

PacBio SMRT technology is based on sequencing by synthesis of circularized DNA created by ligating hairpin adapters to both ends of the target DNA molecule, the read length limit is determined by the template length and the polymerase efficiency (reads up to ~80 kb). All four nucleotides are added simultaneously and measured in real time by florescence; circularization of the template allows to sequence it several times providing a high accuracy (>99.9%; single pass error rate is instead ~15%).

Nanopore sequencing, implemented by Oxford Nanopore (Oxford, UK), is based on a totally different principle. Instead of adding labeled nucleotides to detect their inclusion in a DNA strand complementary to the template, each nucleotide is detected by a nanopore constituted by a mutated form of the *Mycobacterium smegmatis* porin A (MspA; [172]) which serves as a biosensor. A voltage bias generated at the two sides of a membrane produces current which drives ssDNA (or RNA) through the nanopore. An enzyme (polymerase or helicase) is bound to the nucleic acid and works as a ratchet, controlling the step-wise passage of the nucleotides, allowing the detection of the different current perturbation (few pA) generated by each nucleotide when passing through the narrowest part of the nanopore [168]. Reads length limit is mostly determined by DNA fragments length and ultra-long reads, up to almost 1 Mb, were obtained [167]; accuracy can be low (85%, increased to 97% when sequencing both strand) and systematic errors occur in homopolymers [173].

Third generation sequencing technologies performances were compared to 2nd generation technologies using bacterial genomes [172] and were tested alone or in combination with 2nd generation technologies on relevant ascomycetes, as *Saccharomyces* and *Leptosphaeria* species [173,174]. In particular, the hybrid assembly approach—which on one side exploits long reads to obtain high contiguity, and on the other side short reads for high accuracy and error correction- is now becoming the gold standard.

So far (2020), the vast majority of genome is sequenced using at least one Illumina library (86.6%), with a marginal role of the other two relevant 2nd generation technologies: 454 pyrosequencing and IonTorrent, used in 4.8% and 3.2% of the sequencing projects, respectively. Sanger sequencing was used for 1.7% of the genomes and 3rd generation sequencing technologies, such as PacBio and Oxford Nanopore, are becoming extremely popular, and have been used already in 8.2% and 1.9% fungal genome sequencing projects, respectively. The total accounts for more than 100% as many early time projects used second generation technologies in combination with Sanger sequencing, and the same is happening nowadays with second and third generation technologies. In this way accurate chromosome-level genomes can then be obtained in an unprecedented quick and cheaper way than ever.

The huge amount of genomic data produced in the last fifteen years, opens up both great possibilities and challenges; it gives great impulse to the development of bioinformatic tools to assemble and annotate genomes and allows comparative analyses and evolutionary hypothesis testing on genomic scale.

### 3.2. Genome Assembly

Genome assembly refers to the process of putting nucleotides into the correct order to reconstruct the whole genome sequence starting from sequencing reads, which are fragments, usually much shorter than the genome sequence to be reconstructed.

The typical output of NGS Illumina platform are several millions of paired (single for Ion Torrent) short reads (50–300 bp) stored in a text file, usually in fastq format, which associates every sequenced nucleotide with a quality score encoded with a character. Symbols, letters and numbers are used to assure that each nucleotide has a single, unique character expressing its quality; each character then corresponds to a Q-score, which represents the probability of a sequencing error for the nucleotide, and therefore its quality. Quality control and reads filtering is the first step to perform on raw reads before assembling them. The process is automated by softwares, such as Trimmomatic [175], which can perform head and tail trimming on each read, sequencing adapter search and a sliding window scan of the sequence quality, in order to trim the reads when the average quality of any window drops under a user-defined threshold. Quality control of the reads before and after the trimming can be performed by software like FastQC [176], assessing sequences quality, nucleotide composition, duplication level of the reads etc.

The strategy used to assemble a genome from sequencing reads depends on multiple factors. The first thing to consider is the availability of a reference genome or if the assembly must be performed *de novo* (i.e., without knowing the genomic sequence of the target species). Complete or chromosome-level genome assemblies are still a small fraction of the sequenced genomes in Ascomycota, though they are rapidly increasing (1.1% and 14.5%, respectively) 63% of the available genomes are assembled as scaffolds and 21.4% as contigs. Though the majority of genomes are reported as assembled at a scaffold level, many of them seem to have been scaffolded only using short paired reads with small insert length, with likely negligible effects on contiguity from the contig to the scaffold level assembly. Moreover, only a negligible fraction of Ascomycota has been sequenced: ~3.5% of the described species (~57,000; [177]), with an estimated total diversity which is expected to be way larger in Fungi and in Ascomycota too (see Introduction).

**Figure 3:**
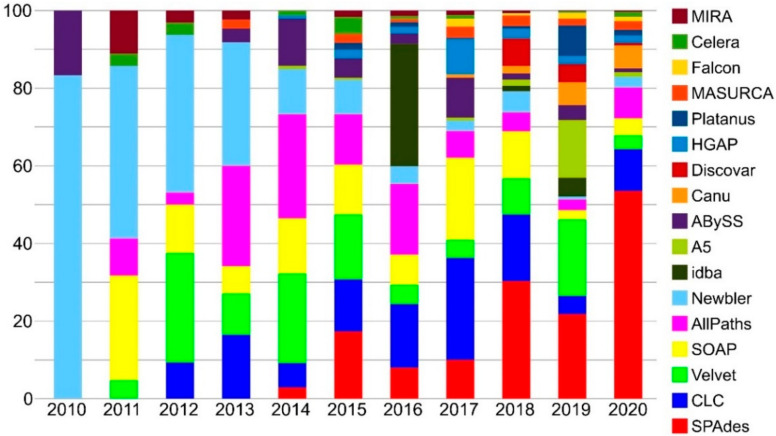
Most used assembly methods by year expressed in percentage. Data were retrieved from NCBI assembly reports, using the ncbi-genome-download script (https://github.com/kblin/ncbi-genome-download/) [148] and a custom script which exploits NCBI Entrez Programming Utilities to retrieve taxonomy. Data for 2020 are only present for assemblies submitted by July 2020. Only tools used for 35 or more genome assemblies are reported. The following tools are not included: SSPACE, SMRT, PILON, SMART, GS, Custom, Gapfiller, Ragout, PCAP, MEGAHIT, Geneious, RaGOO, Phrap, DBG2OLC, Arachne, Roche gs, Albacore, Redundans, HBAR, Minimap, Unicycler, Flye, SeqMan, DNASTAR, MECAT, Casava, Edena, HABOT, Hybrid, Jazz, PBcR, PBJerry, Quiver.

Especially when working with non-model organisms we are mostly dealing with a *de novo* assembly scenario. De novo assemblers use only the reads themselves and their overlap, to solve the genomic “puzzle” without knowing the original picture (i.e., the genome sequence). Different assembly strategies have been developed to efficiently exploit sequences of different length from various sequencing technologies, and to assemble genomes of different size and complexity. Assembly algorithms are classified according to their strategy in three main categories: (i) the Overlap/Layout/Consensus (OLC) methods which rely on an overlap graph, (ii) the de Bruijn Graph (DBG) methods and (iii) the greedy algorithms. Their performances and assembly strategies have been described in an extensive literature [178,179,180,181,182]. In particular NGS technologies based on short reads (up to few hundreds of bp) boosted the develop of assemblers based on DBG. They exploit a directional graph made of k-mers, shorter than reads but still long enough to have specific overlaps, and their connections. This approach allows to manage the enormous amounts of short reads produced by second generation sequencing platforms, reducing their redundancy and finding the path through the graph which produces the most contiguous assembly solution. The perfect assembler, which shall perform the best in every situation and for every genome, does not exist. This is also true for Ascomycota genomes, which vary in complexity and size, although most of them are compact, with an average size of ~37 Mb [183], and relatively simple, if compared to other eukaryotic genomes. Though useful benchmark analyses have been conducted to asses which assembler perform the best in various conditions [180], the most used assembly algorithms are constantly updated to keep up with the sequencing technologies improvement and to solve issues that prevent to get higher quality genome assemblies, such as sequencing errors, repetitive regions and uneven reads depth. The most used assemblers for ascomycetes fungi are reported in Figure 3. Though this information from NCBI assembly report has several limitations, such as the presence of missing data and the update of the same assembler using different versions and pipelines, it still shows effectively some trends in genome assembly. A good example of this evolution is theOLC assembler Celera, which was used during the early years of genome sequencing and is now included in assembly pipeline designed for SMS long reads. Most of the genome to date are still assembled from short reads, with DBG assembler. However, OLC based pipelines suitable for SMS long reads and assembler and able to handle sequences from different sequencing platform (hybrid assembler) are catching on. For the relatively small genomes of ascomycetes sequenced with short reads, SPAdes [184] is by far the most used assembler (Figure 3) and it has been used for more than 1000 assemblies publicly available on NCBI. SPAdes is often able to produce the most contiguous assembly in comparison to other popular de novo assembler commonly used for fungal genomes such as Velvet, [185] or SoapDeNovo [186], and also in comparison to assembler included in commercial tools, such as CLC genomic workbench (data not shown). Its success is determined by the implementation of a multi-sized de Bruijn graph employing different values of k-mers, as shorter k-mers are more suitable for low coverage regions and vice-versa. As mentioned earlier, repetitive structures and uneven sequencing coverage depth are substantial challenges in de novo assembly with short reads. PCR-free SMS long reads can substantially help overcoming these problems, as graph complexity tends to disappear for reads longer than 7 kb, at least in prokaryotic genomes, where most of the repeats are shorter than this threshold [187]. Various approaches have been developed for assemblies of hybrid and long reads only. The principal ones are: (i) SMS long reads only assembly with error correction performed by short reads, (ii) long reads only assembly with OLC approach using high coverage (~50×) to overcome the higher error rate, (iii) scaffolding and gap filling of contigs from NGS short reads using SMS long reads [178].

Up to date, the most used software to assemble ascomycetes genomes using long reads from SMS is Canu, a fork of the OLC Celera assembler [188]. External scaffolder like SSPACE [189] and SSPACE-LongRead [190] can be used to further improve the final assembly. If also a short reads library is sequenced, polishing software like Pilon [191] can be integrated in the assembly pipeline for bases correction. Among long reads only assembler, HGAP [192], also based on Celera, is fairly used for fungal genomes, its hierarchical strategy in conjunction with a single long insert SMRT PacBio library was able to produce high quality bacterial assemblies (~5 Mb genome). Among the hybrid assembly pipeline, a hybrid version of SPAdes (hybridSPAdes, [193]) has been implemented in its last versions. It takes advantage of long reads from PacBio and Oxford Nanopore with a hybrid assembly approach that starts from short reads assembly performed by SPAdes, then completed by means of an aligner and a scaffolder.

After the assembly is complete, coverage and contiguity metrics, as N5, can be assessed by QUAST [194], however, genome completeness is an increasingly used approach to evaluate the quality of an assembly. Pipelines as CEGMA [195], now discontinued, and BUSCO [196] can be used for this purpose. in BUSCO the assembly completeness is inferred on the percentage of universal single copy orthologs retrieved out of the total number of genes searched. Orthologous gene sets are designed at various taxonomic levels with bigger number of orthologs, and therefore a more accurate genome completeness estimation for finer taxanomic levels. These universal single-copy orthologs can also be used for downstream analyses, for instance as training set to improve gene prediction and for phylogenomic inferences [197].

High quality genome assembly can contain complete mitochondrial genome, as mitochondrial DNA is usually abundant, and it is therefore sequenced with high coverage. If a fragmented genome assembly is produced, the mitochondrial genome is still usually covered at a sufficient depth to be completely assembled with software like NovoPlasty [198]. This tool is able toreconstruct complete organelle genomes starting from whole genome sequencing data and a sequence that belong to the target organelleused as a starting seed, which is then extended bidirectionally. Mitochondrial genomes are useful in phylogenetic studies, identification of fungi in food analyses and are the most abundant genomic resources in NCBI GenBank.

### 3.3. Metagenome Assembly

Though most of fungal genomes have been sequenced from axenic cultures, another possible approach is to assemble genomes from metagenomic data. This approach can be particularly useful for organisms that cannot be grown in culture or are associated in a parasitic or symbiotic relationship (e.g., lichenized fungi). Greshake et al. [138] assessed potential of metagenome skimming approach in lichen system aiming at the reconstruction of both symbiotic partner genomes. They developed a benchmark to identify the most suitable assembly methods based on simulated data from already sequenced lichen bionts and then applied them to real-world metagenomic data. They tested both DBG, OLC and assembler specifically designed for metagenomic data. The authors highlighted good potential for this method but also possible pitfalls in real data, especially when coverage of one of the two bionts is lower than the other. The genome of the fungal partner from metagenome had indeed similar completeness compared to the reference genome assembled from culture, though it was less contiguous by one or two orders of magnitude [116]. Best results were achieved for the fungal genome using SPAdes over its metagenomic version (metaSPAdes), IDBA-UD, metaVelvet, Omega and MIRA. This approach has proven very promising especially when applied in conjunction with long-read third generation sequencing [199]. To effectively apply this approach taxonomic binning methods are needed. They use sequence composition, at various levels (GC content, tetramers or longer k-mers) and coverage information to delimit genomes from metagenomic data. Many of them also perform taxonomic assignment using a reference database. Sczyrba et al. [200] performed an exhaustive benchmark of many of these tools, providing useful information to choose the most appropriate tool and narrow the amount of parameters to be tested on a metagenomic dataset. Assemblers and some binning tools, such as MetaWatt [201] and MaxBin [202], were tested. MetaWatt, in particular, recovered most of the genomes from metagenomic simulated communities, while MaxBin performed the best according to precision (purity of the genomes recovered) and recall (their completeness). A general evidence, encountered when dealing with metagenome assembly, is the importance of k-mer selection: small k-mers reconstruct mostly low-abundance genomes, large k-mer favors, instead, high-abundance genome. The use of multiple k-mer assemblers substantially increases the recovered genome fraction [112,200]. The quality of assembly greatly influences the binning step; however, an unsolved challenge is represented by the assembly of closely related genomes, which negatively affects also contigs binning. The use of different classification algorithms, reference, taxonomies databases and data features used for classification (e.g., marker genes, k-mers, coverage) can substantially influence the binning, and the taxonomic profiler performance drops substantially below the family level. Though metagenome investigation tool were mainly developed for prokariotic communties, there are pipelines developed to detect fungi in metagenomes such as FindFungi [203], which uses k-mer based method Kraken ([204], version 2) for reads binning and a custom fungal database. The method also includes detection of false positives based on reads distribution on the reference genome they map to.

### 3.4. Genome Annotation

A genome sequence itself is not very useful, but it starts to become essential when questions on evolutionary relationships or metabolic pathways arise. The process of identifying structures and functions of genes within the genome is called annotation and is key to make a genome fully usable for the scientific community. Though ascomycetes genomes are not among the largest or the most complex genomes within Eukarya, their annotation is not trivial and this is especially truefor non-model organisms. In this context, also the huge effort in human genome annotation still struggles to find a precise intron-exon structure for many protein-coding genes [199], making genome annotation still a challenging task.

The prerequisite for the annotation process is a high-quality, contiguous genome assembly, as highly fragmented draft leads to largely incomplete annotation, that are problematic for downstream analyses. Once this goal is achieved, two strategies, often combined into pipelines, can be used to delimit genes and their structure in a genome: intrinsic (ab initio) gene prediction and extrinsic. The intrinsic approach focuses on information that can be extracted from the genomic sequence itself, the extrinsic approach instead uses the similarity to other sequence type (e.g., transcripts). The intrinsic approach, however, needs well annotated genes used to build gene models and to train the gene prediction software. The more specific is the training set the better the better will work the prediction. The main advantage of the ab initio approach is the possibility to predict species specific genes without extrinsic evidence for them. The extrinsic approach instead can exploit protein databases (e.g., Uniprot) but also transcript information (e.g., from RNAseq), which are aligned to the genome to recover the gene structure. If the alignment is performed with protein sequences, which are more conserved than the corresponding nucleotide sequences, also distantly related sequences can be retrieved, while transcripts can provide more accurate information on gene structure of non-model organism. Genome annotation pipelines usually rely on both approaches starting with an intrinsic prediction, complemented, and eventually overruled, by extrinsic evidence [184]. Though the annotation process usually focuses on genes, genome annotation must deal with repeat sequences: low-complexity sequences such as homopolymeric sequences and transposable elements (TE). TE have an exceptionally variable amount in eukaryotic genomes and though they are usually not particularly abundant in fungal genomes, their amount can vary substantially (from 0.02% to 29.8% of the genome; [205]). A few softwares have been developed to detect and annotate TE, such as RepeatMasker and REPET package [206]; they are often used in genome annotation pipelines as the first stage, before structural annotation of genes. The goal of genome annotation is to assign biologically relevant information to predicted genes, while checking the quality of predicted gene set. This functional annotation is usually performed looking for sequence similarity by BLAST, HMMER or other alignment tools against non-redundant sequence database from NCBI GenBank and UniProt reference clusters. After initial homology search, sequences can be assigned to orthology groups by best-reciprocal or tree-based method [184]. The process of genome annotation can be (partially) automated using pipelines which run batteries of gene finders. An algorithm combines all the evidences and selects the prediction whose intron–exon structure best represents the consensus among the overlapping predictions [207]. A modern annotation pipeline, such as MAKER [208], can use RNA-seq data, combined with alignments to databases of known proteins and other inputs, to find genes and to assign name to many of them.

The inclusion of RNA-seq data is extremely helpful but does not come without caveats: (i) RNA-seq does not precisely capture all of the genes in a genome, some genes are expressed at low levels or in only a few tissues, and they might be missed; (ii) many transcripts expressed in a tissue sample are not genes. Moreover, quality of the assembly can greatly influence the annotation. Fragmented assembly with many contigs of unknown order and orientation can lead to gene number overestimation, while contaminants can introduce genes not belonging to the target organism [199]. Direct RNA sequencing with nanopore sequencing technology can alleviate some of these problems allowing the sequence of full-length transcript and therefore help the annotation. Recently, pipelines exploiting already established tool, have been released. One relevant example is BRAKER1 [209] which combine the resources of two prediction tools: GeneMark-ET [210] and AUGUSTUS [211]. GeneMark-ET performs iterative training and generates initial gene structures. AUGUSTUS uses predicted genes for training and then integrates RNA-Seq information into final gene predictions [209]. BRAKER pipeline has been compared to MAKER2 [212] and, using the genome of *Schizosaccharomyces pombe,* it was also compared to CodingQuarry [213], an annotation pipeline specifically designed for fungal genomes. BRAKER outperformed MAKER2 in the annotation of *Arabidopsis thaliana*, *Caenorhabditis elegans* and *Drosophila melanogaster*. The test on *S. pombe* genome still delivers BRAKER as the best pipeline followed by CodingQuarry and MAKER2 [209].

### 3.5. Methods for Phylogenomic Analyses

Phylogenetic inference arising from molecular data, in the past, predominately relied on one to few ‘traditionally’ used markers, such as ITS, LSU, SSU, COX1, RPB1, RPB2, β-tubulin, MCM7, TEF1-α, γ-actin, ATP6 and CaM [214]. With the advent of genome sequencing, it has become common practice to follow a phylogenomic approach, i.e., to reconstruct the evolutionary relationships of species by utilizing large amounts of phylogenetically informative genomic data.

McCarthy and Fizpatrick [215] reviewed methods used to reconstruct fungal phylogenies up to the kingdom level using genome-based data. They identified four main categories of phylogenomic methods: (i) methods based on a superalignment (supermatrix) of concatenated loci, (ii) methods which reconstruct a consensus supertree from single locus inferences, (iii) methods based on genes content, and (iv) methods based on nucleotide or amino acid composition (alignment-free, AF). The first two are the best-established methods. They both rely on the identification of genetic loci that are orthologous and in single copy in each target genome. This can be achieved using tools like Orthofider [216] or OrthoMCL [217].

Another popular approach is the detection of conserved ortholog genes (COG) using databases such as JGI EuKaryotic Orthologous Groups (KOG) or orthoDB [218], which is used by the BUSCO pipeline. Retrieved loci can be evaluated for phylogenetic signal and substitution saturation prior to phylogenetic inference, as high level of non-phylogenetic signal can lead to highly supported phylogenetic incongruences, in presence, for instance, of a compositional bias in faster evolving third codon positions [219]. Long branch attraction artifacts, especially if the phylogenetic reconstruction method does not account for multiple substitutions, can also take place. In these cases, the evaluation of substitution saturation and the filtering of fastest evolving alignment positions, or even whole markers, can be sometimes advisable. In this context, highly variable sites can be especially detrimental when analyzing deeper phylogenetic relationships because they mostly add noise to the data over the longer time frame considered [220]. On the contrary, the use of only the slowest evolving markers can lead to insufficient phylogenetic signal, leading to less resolution power and lower node support for recent speciation events. Average substitution rates for each marker, or also among branches of a phylogeny or even among sites within each marker alignment, can be evaluated with tools such as PAML [221], while saturation analyses can be conducted with DAMBE [222].

Among the phylogenetic reconstruction methods, the supermatrix approach can take advantage of the statistically powerful maximum-likelihood (ML) and Bayesian approaches. Given an alignment of sequences and a suitable evolutionary model, ML phylogenetic analysis examines all possible trees by their parameters (e.g., topology, site support, branch length) and returns the most likely phylogenetic tree for the alignment. Among the tools commonly used to infer phylogenomic trees under ML we find RAxML [223], which implements fast bootstrapping option, and IQ-TREE2 [224,225], specifically designed for large phylogenies and huge supermatrices. The Bayesian approach is more computational intensive than ML, therefore it is not frequently used yet for large supermatrices.

The second largely used approach to reconstruct phylogenies based on genome-scale data is the supertree approach. It first reconstructs single locus phylogenies, taking advantage of the same methodologies used for concatenated data (e.g., ML, Bayesian), and secondly it adopts methods which aim to reconcile them into a supertree. Different theoretical frameworks such as parsimony, bayesian or the coalescent model, have been implemented. One widely used tool for genome-scale data, that exploits the coalescent to model incomplete lineage sorting and to reconcile gene trees into a species tree is ASTRAL [226]. It uses dynamic programming to search for the tree that shares the maximum number of quartet topologies with input gene trees.

Most analyses based on gene content predates the phylogenomic studies based on supermatrix and supertree approaches because of their computational tractability when compared to these two well- established methods. McCarthy andFizpatrick [215] applied the gene content method, based on a presence-absence matrix of about 13,000 single copy gene families, on 84 fungal genomes. However, parsimony analysis of this matrix highlighted some erroneous placement of branches within Dikarya, in comparison to the other phylogenomic inference methods. Being based only on the presence or absence of genes, the gene content method is by its very nature a “broad strokes” approach and can ignore potentially important phylogenetic information, assuming the same evolutionary history for missing orthologs [215]. Moreover, it requires complete genomes for the comparison to be effectively applied.

Alignment and alignment filtering are crucial steps in classical phylogenetics and their quality can influence the inferred phylogeny. Alignment-free (AF) methods offer an alternative to the assumptions and computational demands of a multiple sequence alignment (MSA), assuming that each species has a characteristic genomic signature that can act as a phylogenetic marker [227]. This signature is usually represented by the frequencies of all the overlapping strings of length k (i.e., k-mers); each genome can be then represented by a frequency vector of length 4k for nucleotides, or 20k for amino acids. A distance matrix is then calculated among the frequency vectors representing the genomes, and a distance-based method can be applied to infer the phylogeny (e.g., Neighbor Joining). These k-mers count-based methods, despite being in their infancy, have proven effective in term of precision and recall when applied on simulated sequences, though they have not been rigorously examined under fully realistic scenarios. So far, they performed well, recovering accurate phylogenies for microbial genomes, and being robust to genome rearrangements. Moreover, they proved to be promising in the detection of lateral gene transfer events [228]. The selection of optimal k-mer size is crucial for the phylogenetic resolution power of these methods and should be chosen according to sequence divergence and length. Longer k-mers exhibits a better resolution for closely related genomes (being more specific), whereas shorter k-mers are more appropriate to identify basal bipartitions. This is reasonable considering that longer k-mers are unlikely to be shared by chance between two (or more) sequences; progressively divergent sequences are likely to share fewer k-mers, as the longest k-mer they share would become shorter and shorter, the more the sequences diverge. These class of methods is fast and scalable to large dataset, an they can be more robust than MSA-based approaches against gene-level evolutionary scenarios, including among-site rate heterogeneity, compositional biases, genetic recombination and insertions/deletions. However, they still retain certain limitations, such as their inaccuracy for relatively short sequences (<10,000 nt) and the typical critical aspects of distance-based methods, which reduce the comparative information to a single number [228]. K-mer frequency method has been implemented in efficient software like Jellyfish [229] and in phylogenomic inference tool such as CVTree [230]. Many AF methods have been benchmarked by Bernard et al. [228] for microbial genomes. Method based on k-mers frequency (discussed above) are robust to divergent input data, in particular co-phylog [231] and D2S [232], outperformed methods based on length of matching k-mers, which instead tend to perform better on highly similar sequence data (where shared k-mers are more common).

In the context of MSA-based phylogenetic methods, reduced representation methods are a valid option to get hundreds (to thousands) of loci to produce large phylogenies or population level studies. This is true even though the sequencing of fungal genomes is now affordable also for small research projects. Many techniques have been developed such as transcriptome sequencing (RNA-seq), restriction site associated DNA sequencing (RAD-Seq) and targeted capture, which can be possibly combined with genome skimming. RADseq was designed to address population-genomic level questions or shallow phylogenetic relationships, while target sequence capture can be suitable for deeper phylogenies. An example of loci targeted for phylogenomics are the ultraconserved elements (UCE). They have been initially identified in human genome [233], later they were also found in distantly related genomes such as insect, worm and yeast [234] but so far they have never been applied in phylogenomic studies of Ascomycota.

## 4. Proteomics Advances in Ascomycota

A list of all reviewed proteomics works is provided in Appendix B.

### 4.1. Opportunistic and Pathogenic Fungi

Initially applied as complementary approach to the analysis of transcriptional changes, proteomics has given a substantial input to the understanding of several aspects of fungal pathogenesis. Investigations of the proteome have helped elucidating the dynamics of important proteins during host–pathogen interaction and disease development [235] and hence has paved the way to the detection of biomarkers of infection and virulence factors [236,237]. Proteomics technologies have thereby also contributed to the improvement of plant and animal protection strategies [238]. First studies using a proteomic approach were carried out on a small number of important phytopathogens—e.g., *Cladosporiu*m sp., *Fusarium* sp.—and on clinically relevant filamentous ascomycetes such as *Aspergillus* sp. [239,240]. Research on fungal pathogens has later intensified due to the growing availability of sequenced genomes. Further, the establishment of standard protein extraction procedures has subsequently incentivized cellular proteomics studies also on non-model organisms [238].

Much effort has been directed to establish reference proteome maps of mycelial, conidial, sclerotial and secreted proteins across a range of species. Several papers reporting comparative analyses of protein profiles have been ever since produced, with latest works increasingly dedicated to the exploration of vesicle proteomics. Profiling methodologies such as classical gel-based methods (i.e., one-dimentional 1D- and two-dimentional electrophoresis 2D-E, SDS-PAGE, GeLC) followed by tandem mass spectrometry (MS/MS), have been for long time the most used approaches for protein separation [241,242]. However, the development of more sophisticated and sensitive high-throughput LC-MS instrumentation has set the stage for in-depth gel-free proteomic investigations i.e., shotgun proteomics, involving the simultaneous analysis of samples in complex experimental designs [243,244]. The recourse to statistic and bioinformatic analyses for the biological interpretation of the results has ever since become a full-fledged part of the proteomic workflows [245].

#### 4.1.1. Whole-Cell and Subcellular Proteomics

A substantial part of the proteomics work in fungal phyto- and zoopathogens has focused on the examination of the “whole-cell proteome”, which is defined as the entire set of all cytoplasmic, sub-cellular and membrane proteins. Generally, the proteome reaction to the mimicking of the in vivo host-pathogen interaction is examined to detect qualitative and quantitative changes in related proteins. This information is utilized to develop a systematic understanding of virulence factors, metabolism and regulatory mechanisms of pathogenic fungi. The elucidation of protein interactions and molecular host–pathogen crosstalk also represent a prolific area of investigation, as well as the study of extremotolerance in the establishment of the infection.

**Figure 4:**
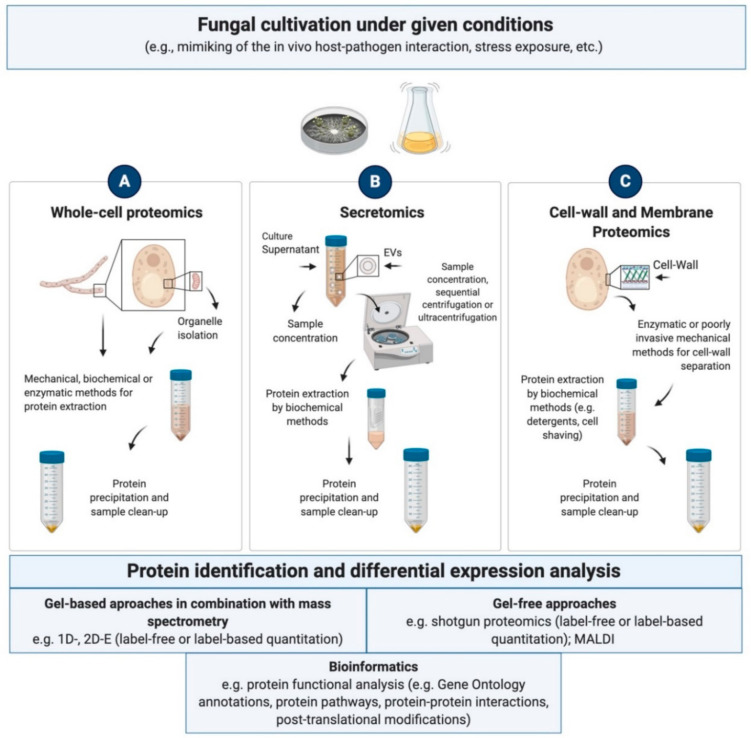
General workflow for fungal proteomics. Steps from cultivation to bioinformatic analysis are shown, with a focus on the three main sample types: (**A**) whole-cell proteome, (**B**) extracellular proteins (secretome) and (**C**) membrane and cell-wall associated proteins. The image was created with BioRender.com.

*Methodological strategies in sample preparation*—The intricacies of whole-cell proteome extraction from fungi are mostly due to the exceptional robustness of the cell-wall. In addition to that, pigments, exopolysaccharides, nucleic acids and lipids are examples of the compounds mostly hindering the isolation of fungal proteins. A number of early studies focused on the development of strategies to overcome this challenge and to ensure an effective cell lysis as well as an adequate release of proteins for reproducible results [246]. Depending on the sample, mechanical, chemical and enzymatic methods are resorted to singularly or in combination (Figure 4A). Independently on the method of choice, since the activity of proteases is reportedly very high in fungi [247], all steps need to be executed in presence of protease inhibitors cocktails to diminish protein degradation [248]. Though the application of enzymatic approaches to the generation of fungal protoplasts has been correlated to better visualized protein patterns [249], sonication, biomass disruption under liquid nitrogen using mortar and pestle or via beating mills represent the most widely used methods for cell disruption [250,251]. In the case of organelles-specific proteins, sub-fractionation and ultracentrifugation steps are required to allow size- and weight-based separation of the intracellular structures [252].

Protein enrichment is achieved by means of organic solvents which favor protein precipitation and also contribute to the removal of contaminants that can interfere with downstream analyses [253]. In the case of pigmented species, the elimination of melanins or carotenoids requires special effort. Melanins and carotenoids are long chain polymers from phenolic or indolic precursors with several functions in cell protection and virulence, which accumulate in the cell-wall contributing to a thick and rough cell surface [254]. Precipitation with trichloroacetic acid (TCA) and acetone or a combination of the two is most often used for fungal protein extracts, followed by pellet washes with methanol and/or acetone [31]. Zwitterionic detergents, urea and thiourea are generally applied to ease protein solubilization following TCA-treatment.

Sample-tailored adjustments of this basic workflow are often required and may deal with slight alterations in buffer composition or with more radical changes to the protocol. As no single protein extraction procedure is able to capture the whole proteome, the method of choice should reflect the research objectives and help to successfully overcome sample-related challenges. Sample preparation approaches are also evaluated based on their compatibility with the techniques used for protein separation and analysis. Special precautions are required by studies in vivo, e.g., in planta, due to the need to separate and discriminate cells from the pathogen and the host and their respective proteomes. In this respect, the advent of bioinformatic tools has greatly facilitated the analyses and increased the accuracy of the data.

*Collection of proteomics works*—A range of proteomics techniques has been extensively deployed to obtain comprehensive representations of the proteome with a focus on disease development, virulence factors and antifungal agents. Several studies have concentrated on medically relevant yeasts such as *Paracoccidioides* sp. and *Candida* sp., whose infectious behavior involves the formation of well-structured biofilms to safeguard the pathogen from the host defense mechanisms. Recently, Chaves et al. [255] used the Nano Ultra performance LC-MS Elevated Energy (NanoUPLC-MSE) technology to analyze the proteomic response of the highly virulent *P. brasiliensis* Pb18 following interaction with macrophage cells, the first line of defense against paracoccidioidomycosis (PCM) during lung infection. The study, which used both INF-γ-primed and non-primed macrophages, helped elucidating that intracellular environment of non-primed macrophages is more permissive to the survival and multiplication of *P. brasiliensis*, thereby substantiating the finding that the fungus is affected by activation of phagocytes with INF-γ. Pathways described as essential for the survival of pathogens inside macrophages (i.e., phosphate pentoses pathway, synthesis of cell wall components and mitochondrial activity) were in fact observed only or with higher intensity in yeast cells recovered from non-primed macrophages. For a better understanding of the molecular underpinnings of host-fungus interactions, Do Amaral et al. [256], carried out a quantitative proteomic study (2D-E, LC-MS/MS) in an attempt to correlate expression profiles to in vivo virulence of a number of *P. brasiliensis* strains isolated from environmental and animal sources, including Pb18. Highly virulent isolates that caused disseminated disease in a murine model of PCM were found to overexpress proteins related to pathogenicity, metabolism and redox homeostasis (e.g., glutathione reductase, peroxisomal catalase, RNA binding protein), indicated as critical in regulating the fugus ability to escape the host immune system. In an attempt to detect compounds with antifungal activity, Muthamil et al. [257] showed the inhibitory potential of oleic acid on biofilm formation and virulence of *Candida albicans.* 2D-E in combination with matrix-assisted laser desorption ionization-time of flight (MALDI-ToF/ToF) MS, indicated that oleic acid triggers an oxidative stress response and significantly down regulates the expression of enzymes (e.g., acetyl-coA hydrolase, alcohol dehydrogenase) which play a key role in the fungus yeast-to-hyphal transition. These results were substantiated by in vitro virulence assays.

In phytopathogenes, proteomics studies of host-pathogen interaction revealed that ROS detoxification and production of cell wall degrading enzymes are crucial for successful colonization of the plant by the fungus. Plant defense mechanisms on the other hand, may include cell wall reinforcement and remodeling, production of pathogenesis-related proteins and the generation of a stressful apoplastic environment [258]. A few studies explored the functions of post-translational modification (PTM) during infection. Chen et al. [259], employed a label-free quantitative proteomics analysis to unravel the regulatory mechanisms of N-glycosylation—in which a glycan moiety is added to the amide group of an asparagine residue—in the rice blast fungus *Magnaporthe oryzae*. The regulated N-glycosylated proteins, nearly all key components of the endoplasmic reticulum quality control (ERQC) system in conidium and appressorium (i.e., Gls1, Gls2, GTB1 and Cnx1), were found to be involved in the coordination of different cellular processes for mycelial growth, conidiation and invasive hyphal growth. Lysine succinylation was instead analyzed in anotherpathogen of rise blast disease, *Pyricularia oryzae,* by Wang et al. [260]. Here, LC-MS/MS combined with a high-efficiency succinyl-lysine antibody revealed that succinylation occurs on several key enzymes of the tricarboxylic acid cycle and glycolysis pathway as well as on pathogenicity-related proteins—e.g., heat shock protein (SSB1) and a subtilisin-like proteinase (SPM1)—indicating that such PTM may play important roles in both the regulation of basal metabolism and infection. These findings indicate that PTM studies hold a great potential for the control of plant diseases that represent a great threat to world food security.

Studies of potential biomarkers of drug action were carried out in several opportunistic and pathogenic species. Cagas et al. [261] used isobaric tagging for relative and absolute quantitation (iTRAQ)-based proteomics to explore the cellular processes affected in *Aspergillus fumigatus* mycelia following exposure to caspofungin and found the levels of a number of proteins and potential allergens, including the cell wall chitinase ChiA1 and the cytotoxin AspF1, to be decreased. Using 2D-E in combination with IgE-immunoblotting, more than 20 allergenic proteins of fungal spores could be identified in the airborne mold *A. versicolor* (e.g., glyceraldehyde-3-phosphate dehydrogenase, catalase A, enolase) and a component-resolved allergen testing could be obtained using patients’ serum IgEs [262]. Shi et al. [263] used iTRAQ to characterize the biological actions of wuyiencin treatment on the grey mold *Botrytis cinerea*, common cause of plant disease, and found evidence of this biological control agent to debilitate the growth and pathogenicity of the fungus. Both ultrastructural alterations and changes in protein abundance were observed and the latter concerned the downregulation of proteins involved in amino acid, protein and nucleotide biosynthesis, a phenomenon frequently observed also during starvation of pathogenic fungi. Conversely, carbohydrate metabolism and cell wall stabilization-associated hydrolases—hallmark of nutritive restriction and the fungus recourse to alternative carbon sources—were found to be upregulated. In a similar fashion, Aumer et al. [264] resorted to complementary mass spectrometry approaches (i.e., MALDI and LC-ESI-MS/MS) to identify target proteins and pathways of the antifungal peptide ETD151 homologous to insect defensin, in *B. cinerea*. The effects of ETD151 on both membrane’s integrity and protein composition were demonstrated by microscopy and differential expression analyses respectively. Unlike what observed for wuyiencin, ETD151 did not interfere with carbohydrate, protein and lipid biosynthesis, however it triggered the activation of part of the MAPK pathway, involved in filamentous growth, cell wall integrity (CWI) and the high osmolarity/glycerol (HOG) response. These results confirmed what previously reported for other plant defensins (e.g., RsAFP2) in *C. albicans* [264].

Proteomics of zoo- and phytopathogens also encompasses proteome profiling studies. An analysis of the conidial proteome of *A. fumigatus* was performed by Anjo et al. [265] applying Sequential Window Acquisition of All Theoretical Mass Spectra (SWATH-MS). A time course evaluation of the proteome revealed how the metabolic state of conidia switched from very active to dormant as the availability of nutrients decreased during cultivation. The increased levels of hydrolytic enzymes towards the end of cultivation in 30-days old conidia went hand in hand with cell autolysis [265]. A comparative study of conidia and mycelial proteome was carried out in the entomopathogenic fungus *Metarhizium robertsii*, widely applied for biological control [266]. iTRAQ-based proteomics revealed proteins differential regulation in the two developmental stages, with higher levels of stress-related proteins (i.e., peroxisome pathway) and proteins involved in biosynthesis of secondary metabolites and glyoxylate and dicarboxylate metabolism, in conidia. These pathways were indicated by the authors as crucial for conidial dormancy and efficient germination but also for stress resistance. Gel-based approaches were used to elucidate the protein repertoire in the thermally dimorphic opportunistic fungus *Talaromyces marneffei* (syn. *Penicillium marneffei*) [267,268]. Following comparison of yeast and hyphal cells proteomes, a number of proteins (e.g., catalase-peroxidase, cytochrome P-450, Hsp90 and isocitrate lyase) were reported to be expressed more abundantly in yeast cells. Secreted glyceraldehyde-3-phosphate dehydrogenase and Hsp60, identified as upregulated in mycelium and yeast phases respectively, were suggested as adhesion factors for pathogen-host interaction, while polyketide synthases, involved in melanin biosynthesis, was indicated as virulence factor [268]. More recently, Otun et al. [269] used 1D-E and mass spectrometry to profile and characterize the mycelial proteome of the necrotrophic phytopathogenic fungus *Sclerotinia sclerotiorum*. Protein functional analysis applied to the 1471 identified proteins, resulted in the first map of protein networks in this species and revealed the presence of proteins reportedly involved in virulence and synthesis of mycotoxins like tripeptidyl-peptidase and ochratoxin [269].

Other studies have sought to explore the effects of environmental factors and restraining cultivation conditions on fungal growth and pathogenicity. Comparative 2D-E was applied to clarify the role of zinc during infection in *Paracoccidioides* sp. [270]. Gel protein profiles of the ascomycetous yeast cells grown under zinc rich conditions and during a 24 h zinc starvation, were analyzed. Rearrangements in the proteome, also corroborated by RT-qPCR, revealed oxidative stress as the major cellular response induced during zinc withdrawal. 2D-E coupled with MALDI-MS/MS was used to further characterize *Paracoccidioides* sp. oxidative stress and reported a global activation of antioxidant proteins (e.g., catalase, superoxide dismutase (SOD), cytochrome C peroxidase (CCP) and thioredoxin) to protect the yeast cells from both short and long term H_2_O_2_-treatment [271]. Da Silva Rodrigues et al. [272] applied UPLC-MSE to explore proteins differential expression during osmotic shock in *P. lutzii*. The results of this study indicated that the response to osmotic stress helps the fungus to deal with the host environment during dissemination to organs and tissues. Proteomic reactions to carbon limitation were moreover investigated in *Paracoccidioides* spp. to mimic adaptation to host during infection and suggested a metabolism reprogramming during carbon starvation in favor of gluconeogenesis and ethanol production [273]. Additional investigations of *P. lutzii* and *P. brasiliensis* were carried out to shed light on the survival mechanisms of human pathogenic fungi towards nitrosative stress, the main defensive strategy applied by immune cells during infection [274]. Proteins showing increased abundance were related to lipids and amino acid metabolism as well as to the oxidative stress response (i.e., SOD and CCP). Further aspects of the infection were examined in *P. brasiliensis* by Parente-Rocha et al. [275]. The proteomic response of the dimorphic fungus to macrophage internalization was investigated and, similarly to what observed for nitrosative stress defense, SOD and CCP and thioredoxins were upregulated. Other proteins positively regulated were involved in cell rescue, defense, virulence and amino acid catabolism. Proteins related to glycolysis and protein synthesis were instead downregulated, this suggesting that *P. brasiliensis* adapts to the macrophage milieu by reprogramming its metabolism to enhance protection against oxidative stress and to cope with glucose deprivation.

In a similar attempt to get an insight into the mechanisms at the base of pathogenicity and ecological plasticity, the influence of temperature stress was examined on the proteome of the thermotolerant and opportunistic black yeast *Exophiala dermatitidis* [276]. Here, 2D- Difference Gel Electrophoresis (DIGE) LC/MS-MS showed the lack of a typical stress response upon supra- and suboptimal temperature and no protein differential expression following short-term (i.e., 1 h) exposure to temperature stress. A general reduction of the metabolic activity, as well as rearrangements at the cell wall and cell membrane levels, were instead recorder after long-term (1 week) exposure to cold. These results suggest that a fine-tuning modulation of proteins is an energy-saving mechanism at the base of *E. dermatitidis* adaptation to altered growth conditions. Deng et al. [277], sought to deepen the knowledge of the heat shock response (HSR) mechanism in *Aspergillus niger* and analyzed the fungus intracellular proteome under temperature stress (50 °C) using both iTRAQ and label-free shotgun proteomics. Some of the protein pathways found to be increased in the treatment as compared to the control condition (30 °C), were involved in protection and repair functions, e.g., oxidative phosphorylation and the citrate cycle (TCA cycle), possibly involved in the maintenance of cellular energy homeostasis, and the porphyrin and chlorophyll metabolism, which directly affect the scavenging of heat-induced reactive oxygen species (ROS). Oh et al. [278], used unfractionated gel-free LC-MS/MS to elucidate the mechanisms of tolerance to limited nitrogen availability in *Magnaporthe oryzae*. Pathways increased under nitrogen starved conditions included nitrogen catabolite repression, melanin biosynthesis, protein degradation and protein translation. The increase in melanin production in particular, known to be essential to *M. orzyae* successful penetration into the host, is consistent with observations of a link between nitrogen starvation and the development of pathogenicity. Jacobsen et al. [279] used label-free quantitative mass spectrometry to gain insights into the response to osmotic stress (1M NaCl) in *C. albicans*. These data shed light on adaptations that contribute to *C. albicans* pathobiology and indicated that survival to salt stress involves a transient reduction in ribosome biogenesis and translation, together with the accumulation of glycerol, a key osmolyte for this yeast. Still in *C. albicans*, Ingle et al. [280] investigated proteins specific to chlamydospore, a unique non-virulent morphological form induced in response to stresses and occasionally found in tissue samples. SWATH-MS analysis revealed morpho-physiological changes in cellular architecture such as cell wall thickening, that enable survival under hostile micro-environments. Similarly, the upregulation of glycolysis, TCA cycle, glyoxylate and fermentative pathways during chlamydosporulation should facilitate persistence of *C. albicans* under glucose limiting and microaerophilic condition.

#### 4.1.2. Secretomics

The secretome, i.e., the extracellular proteome, describes a cohort of protein entities released by living organisms [281]. Secreted proteins—either freely released or associated with outer cell wall—are involved in cell-to-cell communication and in the exchange of information with the environment [282]. Depending on the organism lifestyle, the fungal secretome plays important roles in the degradation of organic material and consequent nutrients acquisition, or in symbiotic and pathogenic relationship with the host. The secretion of extracellular vesicles (EVs) has been observed in a number of species of melanotic fungi and yeasts [283] and recent findings have revealed a role for fungal EVs in triggering anti-microbial activities as well as in modulating virulence strategies [284]. Hence, investigations of the secretome and its regulation under a given set of conditions are crucial to shed light not only on the cell basic metabolism and the organisms’ life cycle but also in stress survival and pathogenesis in view of the development of antifungal therapies.

*Methodological strategies in sample preparation*—Secretory proteins are commonly isolated from the cultivation media after filtration or centrifugation to separate the fungal biomass from the culture supernatant. Proteins are subsequently extracted through precipitation steps akin to those applied to whole-cell protein samples. As unhindered as it may seem when compared to the more effortful strategies deployed to the separation of membrane and intracellular proteins, this workflow is not entirely free from pitfalls. The main challenge is to differentiate secretome from proteins not originating from the extracellular repertory, e.g., those released by endopeptidases during cultivation or following cell death [285]. In this respect, a number of bioinformatics tools are useful to discriminate intracellular proteins from proteins that are ultimately destined to the secretory pathway or to the cell membrane based on signal peptides [286]. Other types of contaminants may include pigments and polysaccharides; in order to prevent their interference with downstream procedures for protein analysis, precipitation and sample clean-up steps are generally required. Melanin can be found in culture fluids in the form of granules, which confer the culture supernatant a dark-brown coloration [287]. It also represents a major obstacle to proteomic analyses of EVs from melanotic fungi, as it may be present both in the media and inside vesicles that export melanin extracellularly [284]. Proteomics of EVs entails vesicles purification from cell-free culture supernatants and generally requires sample concentration by membrane ultrafiltration followed by diverse separation techniques—e.g., sequential centrifugation and ultracentrifugation or density gradient centrifugation, filtration, immuno-affinity, etc. [288]—that can be used individually or combined. Protein extraction from purified vesicles is achieved solely via biochemical interventions with e.g., chaotrope-based homogenization buffers or precipitation [24] (Figure 4B). As the thorough isolation of pure fractions and the integrity of the sample are both crucial, further purification steps in HPLC can be performed to aid the removal of non-exosomal material prior to protein extraction [289]. However, thorough clean-up procedures together with the limited sample amounts, may often result in low recovery of EVs and of exosomal proteins [290].

*Collection of secretomics works*—Though initially limited to a few biotechnologically relevant species exploited for protein production, fungal secretomics has grown additional relevance ever since extracellular proteins were identified as the main effectors responsible for pathogenic or symbiotic interactions between fungi and their plant or animal hosts [291]. Secretome proteomics studies have hence elucidated the infection strategy of several species of zoo- and phytopathogenic fungi, through the identification of virulence factors and pathogenicity related proteins (PRP). First investigations of the secretome in *B. cinerea* brought to light enzymes with roles in the degradation of plant defensive barriers (e.g., pectin methyl esterases, xylanases and proteases), which were recognized as virulence factors [292]. More recently, a comparative analysis of *B. cinerea* parental strain with mutants exhibiting total loss of virulence, confirmed the role of proteases and carbohydrate-active enzymes (CAZymes) in host invasion by degradation of the plant cell wall, since these enzymes appeared down-accumulated in the mutants [293]. Monteiro et al. [294] used a proteomic approach to uncover hydrolythic enzymes in the secretome of *Trichoderma harzianum*, a cellulose degrader also known to be a mycoparasite of several plant pathogenic fungi. The results led to the conclusion that secretomes related to mycoparasitism and cell wall degradation are entirely different [294]. pH-dependent changes of the secretome were investigated by Li et al. [295] in the fruit pathogen *Fusarium proliferatum* using 2D-E-MALDI-TOF/TOF-MS. A correlation was found between high pH values and the downregulation of several cell wall degrading enzymes (CWDEs). Based on these results, the role of 1,3-β-glucanosyltransferase, SPR1-exo-1,3-beta-glucanase, glucan 1,3-beta-glucosidase and gluconolactonase as pathogenicity factors was confirmed and a decrease in CWDEs was associated to a decrease in the fungus pathogenicity. More recently, a shotgun label-free approach combined with systematic analysis served the identification of novel effectors in the secretome of *F. oxysporum* (f. sp. *cubense*), causal agent of wilt disease in banana fruit [296]. The predicted effector candidates, showing regulation at different stages of the infection under simulated in planta status, included the cysteine rich SIX (secreted in xylem) effectors which was also identified in other *F. oxysporum* species. Protein effectors associated with barley infection were just recently observed for the first time in the secretome of the fungal pathogen *Pyrenophora teres* [297]. The candidate effectors were speculated to have a role in chitin binding and in suppression of both host recognition of the pathogen and host cell death.

Reports of effector-type proteins also include secretome studies of entomopathogenic fungi, where effectors specific to different insect tissues have been often observed in conjunction with large secretomes [298]. Large secretomes possibly reflect the many habitats pathogenic fungi must adapt to in insects, as also seen in several isolates of the saprophyte and entomopathogen *Beauveria bassiana* [299]. A number of insect- or host cuticles-modulated exoenzymes (e.g., chitinase D), whose activity was assessed, were proposed by the authors as bioinsecticidal. Some overlapping in the secretion of exoenzymes was found between the secretomes of *B. bassiana* and that of the pest control agent *Metarhizium anisopliae*, where classical fungal effectors (e.g., cuticle-degrading subtilisin Pr1A, B, C, I, J proteases, ROS-related proteins and oxidoreductases) were detected as upregulated following exposure to host cuticles [300].

In human pathogens, secretome investigations aiming at the detection of proteins essential for infection include the recent work on *Candida glabrata* [301], one of the causative agents of nosocomial bloodstream infections (BSIs). The authors used LC-MS/MS to delineate the role of CgYapsins (11 glycosylphosphati-dylinositol-anchored aspartyl proteases)—reportedly involved in virulence—in interaction with host cells. A comparative analysis of wild-type and of an avirulent Cgyps1-11Δ mutant strain revealed a mutant secretome 4.6-fold larger than that of the wild type (119 proteins), this suggesting that CgYapsins may modulate the secretome of *C. glabrata*. Eight CgYapsins were identified in the wild-type and the cellular release of two (i.e., CgYps1 and CgYps7) was confirmed by Western analysis. In the opportunistic human pathogen *A. fumigatus,* pathogenesis-related proteins were detected as accounting for 27% of all the exrtacellular proteins identified by LC-MS/MS. Secretion of these proteins was correlated to that of glycoside hydrolases and proteases, altogether contributing to the colonization of the host [302].

Studies of the extracellular proteome also encompass the analysis of EVs. Remarkably, most of the proteins found in fungal vesicular fractions lack the characteristic signal peptides required for conventional secretion [303], thereby the origin of EVs from unconventional or still unknown pathways of secretion can be hypothesized. The comparison of EVs protein profiles from opportunistic species further revealed overlapping in protein composition, which suggests the presence of signature proteins with clear roles during infection [283]. The ubiquitous environmental fungus and opportunistic human pathogen *Alternaria infectoria* was the first filamentous fungus to be described secreting EVs. Analysis of their content revealed the enzyme polyketide synthase, which catalyzes a melanin intermediate and is associated with pathogenesis in various fungi [304]. Similarly, proteins involved in synthesis of polyketides represented, together with proteases and proteins that function in basic cellular processes, the majority of proteins packaged into the EVs of the cotton pathogen *F. oxysporum* f. *vasinfectum* [305]. A total of 206 vesicles proteins were identified in the fatal human pathogen *Histoplasma capsulatum* [306], some of which had key roles in the onset of pathogenesis and host immune responses (i.e., glyceraldehyde-3-phosphate dehydrogenase, histone H2B, Hsp60) and were immunogenically active. Hsp60, one of the major hits in *H. capsulatum* EVs, was shown to interact with the macrophage cell surface and to have a key function in allowing the cells to be internalized and evade the macrophage defense. In addition, Hsp60 is considered an immunodominant antigen that orchestrates the adaptation to temperature stress [307]. This and several others vesicle proteins were speculated to have moonlighting functions and thereby to be involved in multiple activities, which enable the cells to efficiently perform diverse tasks despite limited genomes [308]. Remarkable alterations of fungal EV protein composition and release were recently observed in *H. capsulatum* based on the available nutrients for fungal growth, which also suggests that different host and environmental conditions may potentially lead to a more or less virulent state of a fungus [309]. A comparative proteomics analysis of vesicle and vesicle-free fractions was performed in *Paracoccidioides brasiliensis* yeast phase and resulted in the identification of 205 and 260 proteins, respectively, with 120 overlapping both fractions. Similarly to what observed in *H. capsulatum*, EVs-associated proteins included sequences mostly related to response to stress (i.e., HSPs), oxidation/reduction (e.g., proteins that could neutralize host defense mechanisms), and carbohydrate and protein metabolism (i.e., lipases and proteases to help fungal dissemination) [283]. EVs proteome has also been partially characterized in the dimorphic fungi and causative agents of sporotrichosis *Sporothrix schenckii* and *S. brasiliensis*, where the presence of immunogenic components and proteins associated with metabolism and transport (i.e., HSPs, serine/threonine protein kinases, cell wall glucanases) that could also act as virulence factors, was observed [310].

#### 4.1.3. Cell Wall and Membrane Proteomics

Cell wall (CW) and cell membrane-associated proteins play critical roles in both the ecophysiology of fungal organisms and the host-pathogen interaction. Being integrated in membrane structures, they are part of protective layers sheltering the cell from external disturbances and changes in environmental conditions [311]. CW protein composition is quite dynamically dependent on the growth phase. Cell wall proteins (CWP) are actively involved in cell-to-cell first contact and communication as well as in biofilm formation and adhesion to host cell tissues or to abiotic compounds. Interestingly, the fungal CW is composed of molecules that are mostly absent in humans, hence, it represents a quite attractive target for the development of safe antifungal drugs to combat fungal infections [312]. Strategies for the investigation of membrane and wall proteins are therefore being increasingly applied to the study of opportunistic and pathogenic fungi in order to elucidate the role of surface-exposed proteins during infection and to detect target molecules for host immunity.

*Methodological strategies in sample preparation*—The analysis of CW and membrane associated proteins requires the separation of the wall from the protoplast, which can be achieved with or without resorting to cell disruption. In order to prevent release of cell content and consequent contaminations, poorly invasive methods for the mechanical breakage of the cells such vortex, sonication and washing steps with buffers with ionic strength, are generally preferred [313,314]. Enzymatic treatments can otherwise be performed [315] (Figure 4C). As for whole cell proteome extraction procedures, detergents, denaturing and reducing agents can be used, however they are most effective for the isolation of proteins non-covalently incorporated into the wall. Cell incubation in DTT-based homogenization buffers for instance, has been shown to help protein extraction in black yeasts without affecting membrane integrity and thereby the release of cytoplasmic and membranous contaminants [315]. In case of proteins associated with cell wall polysaccharides, hydrolytic enzymes and chemicals are often additionally required [316]. Based on the nature of the protein linkage to the wall—i.e., alkali sensitive linkage (ASL)-, GPI-linkage, chitin-bound proteins—different types of commercially available enzymes like e.g., β-glucanases and chitinases or alkali and acids can be applied [24]. Acids are often used to promote protein deglycosilation prior to downstream separation techniques. Alternatively, “cell shaving” is performed by the incubation of intact cells with proteolytic enzymes to ensure protein cleavage and the release of peptides, which can thereafter be identified by mass spectrometry [317].

*Collection of proteomics works*—As a result of the availability of complete genome sequences, cell wall proteomics has developed quickly as a line of research, with the majority of works hitherto focusing on ascomycetes. The most extensive analyses of fungal cell wall proteome were performed on *C. albicans*. Hernáez et al. [318] and Vialás et al. [315] elaborated techniques for the analysis of the *C. albicans* cells existing in the unicellular forms or in the form of hyphae and biofilm. Hernáez et al. [318] reported the identification of over 30 proteins with known function using MALDI TOF/TOF, the majority of which were involved in the cell wall organization and biosynthesis (i.e., cell wall mannoproteins, chitinases and endo-glucanases). The remaining proteins played a role in general cellular metabolism, transport, protein fate and fungal defense and virulence (e.g., HSPs Ssa1 and Ssb1). Subsequently, the study of Vialás et al. [315] indicated the presence of distinctive proteins in *C. albicans* yeast-like cells, hyphae and cells involved in biofilms formation. Proteins shared by all morphological forms included, among others, the chitinase Cht3 (responsible for cell wall maintenance), Hsp70 and Ssa2, described as non-covalently bound adhesin-like molecules. By contrast, GPI anchor proteins covalently linked to polysaccharides such as phospholipases were identified at the surface of yeast-like cells, while typical adhesins in the cell wall of hyphal forms. Comparative studies of the cell wall fractions from mycelia and yeast were also performed in *Paracoccidioides lutzii*, where cells fractionation coupled to NanoUPLC-MSE revealed 7 predicted GPI-dependent cell wall proteins potentially involved in cell wall metabolism and adhesins such as enolase and glyceraldehyde-3-phosphate dehydrogenase, previously described in *Paracoccidioides* spp. [319]. Ecm33, and proteins like glucanase Crf1 were instead found to be common to both fungal phases [320].

A proteomic analysis of the cell wall in response to oxidative stress in *Sporothrix schencki*i [321] elucidated the strategy used by the fungus to cope with ROS and evade the phagocytic cells from the host. The proteins upregulated and potentially involved in the mechanisms of oxidative stress response included, also in this case, GPI-anchored cell wall proteins, covalently linked cell wall proteins, Hsp30, lipases, chitinases, EglC, along with thioredoxin1 (Trx1) and SODs. The involvement of cell wall proteins in virulence, adhesion, and resistance to oxidative stress was demonstrated in *C. albicans* in the frame of a tandem-MS comparative proteomics study involving a Pga1 (Putative GPI-anchored protein 1) null mutant lacking a cell wall protein. The authors showed that the mutation affected the aptidude for adhesion and biofilm formation and influenced the expression of cell surface proteins like the antigenic determinant and virulence factor Hsp 90, aspartyl proteases, septins, Int 3 and members of the lipase family, which were detected exclusively in the wild type [322]. Novel cell wall proteins with a critical role in infectionhave been recently detected in *A. fumigatus* conidia through LC-MS/MS-based cell wall proteome profiling. One protein, designated as conidial cell wall protein A (CcpA), was recognized the role of conidial stealth protein by altering the conidial surface structure to minimize innate immune recognition [323]. Other conidial proteins were identified by a combination of cell wall shaving and hydrogen-fluoride (HF)-pyridine treatment, the most abundant of which was a RodA surface hydrophobin. RodA forms a cohesive, proteinaceous layer on resting conidia to increase fungal survival and to mask host immune responses [324].

Altogether, the proteomic comparison of the surface proteins in clinical isolates of *Aspergillus*, *Candida*, *Cryptococcus*, *Coccidioides* spp., etc. revealed highly expressed conserved cell wall proteins across the species, which have been suggested as potential cross-protective vaccine candidates. The candidates include 1,3-β-glucanosyltransferases (Bgt1, Gel1-4), glucanase Crf1, Ecm33 and endoglucanase C (EglC) [249], among others. These data substantiate what previously reported for *A. fumigatus* and *Coccidioides posadasii*—the causative agent of coccidioidomycosis—following label-free quantitative MSE of the cell wall fraction from two different media [325].

### 4.2. Proteomics Advances in Extremophilic and Extremotolerant Fungi

The proteomic investigation of extremophiles has been hitherto prevalently aimed at the understanding of how these organisms persist at life-threatening ecological conditions. By means of different available approaches, light was shed on the protein expression profiling under given stress conditions. Functional and structural characterization have on the other hand been targeted to uncover crucial modifications at the base of stability and activity of proteins under extreme values of physicochemical parameters [326]. Though unravelling adaptive mechanisms has been a major focus, proteomics of the extremophiles also plays a significant role in the field of biotechnology as it can directly contribute to the screening for proteins and enzymes of interest. In this respect, extremotolerant and extremophilic fungi have gained increasing attention as natural sources of novel molecules and compounds, especially in view of the advantages offered by their products over less tolerant counterparts [39]. Hence, the study of their proteomes may also aid the development of strategies at gene and protein level ultimately leading to biotechnological innovations.

Throughout the last decade the number of proteomics studies focusing on stress-resistant fungi has increased significantly and reached over less-known species, thereby raising our comprehension of the system biology of those organisms to a higher level [48]. The knowledge at the functional proteomic level is however far from being thorough, mainly due to the effortful application of protein techniques to extremophiles [24]. The lack of proteomic data for several extremophilic and extremotolerant species, indeed indicates major analytical and methodological challenges especially concerning sample preparation [327]. As a result of the adaptation to harsh living conditions, proteins have evolved a range of specific features—e.g., thermo-stability, low water-solubility, altered surface charges, resistance to chemical denaturation—that interfere with the extraction and separation procedures [328].

A fine example of methodological difficulties posed by the sample is provided by the number of melanized microfungi and yeasts grouped under the name of black fungi and black yeasts. From a proteomics perspective, the repertoire of morpho-physiological and cellular adaptations supporting these organisms’ stress survival—e.g., multi-layered cell wall encrusted with melanin granules, pigments, polysaccharides and lipids [329]—represent major obstacles to cell disruption, protein determination and separation. As low protein yield and high content of impurities represent the main issue toward the proteomics of extremophilic fungi, the analysis of their protein profiles necessitates optimization and a case-by-case assessment of the methods for sample preparation and analysis [24].

#### 4.2.1. Whole Cell and Subcellular Proteomics

Much of the proteomics research work hitherto produced in the field of extremophilic and extremotolerant fungi deals with the investigation of the “whole-cell proteome”. Various proteomics techniques are selected for survival and adaptation study where changes in protein abundance under environmental or simulated conditions are monitored. Following exposure to different physical and chemical stressors, comparative analyses of control and treatment conditions or of wild type and mutant organisms are carried out to identify e.g., protein groups involved in the stress response and the effects of a given experimental condition on the protein repertoire.

*Methodological strategies in sample preparation*—The isolation of the whole-cell protein content from extremophilic fungi often requires additional steps to the standard procedures used for less recalcitrant samples and generally both mechanical breakdown of the cells and biochemical interventions are necessary (Figure 4A). The use of beating mills in association with glass or zirconium beads, or alternatively of mortar with pestle, has proven to be a successful prerequisite to the disruption of fungal biomass when dealing with multi-layered and melanized cell walls [330,331]. Freeze-drying or lyophilization of the biomass are in some cases applied prior to the milling procedureto aid cell breakage. As an extensive milling is generally required for the thorough breakage of the cells, performing this step at low temperatures is paramount in order to reduce protein degradation by intracellular proteases or protein fragmentation and oligomerization [332]. To enhance the disintegration of cell walls, mechanical cell disruption is frequently combined with homogenization buffers containing chaotropes (e.g., urea and thiourea) and non-ionic detergents like CHAPS [24]. Although harsh treatments are often necessary for cell disruption, they may easily jeopardize the organelles state; hence, protocols for protein extractions shall be accurately selected and adapted to the specific goal [242].

The removal of interfering compounds is another critical step on the way to obtaining a high-quality protein extract. Pigments, mainly melanins, are found in great abundance in several extremophilic and extremotolerant fungi and their removal is especially challenging in view of the fact that melanin reportedly binds to proteins [333]. The recourse to phenol-based methods for protein extraction followed by precipitation steps represents a common strategy for the reduction of melanin’s interference, yet not always completely successful. Due to the solubility of polyphenolic compounds in organic solvents such as phenol [334], the co-extraction of melanin and proteins may occur. Furthermore, melanin and proteins co-precipitate in presence of organic solvents, thus often resulting in darkly pigmented protein pellets [24]. As a measure to counteract this problem, the use of charcoal powder by direct addition to the homogenization buffer has been suggested. While quite useful to reduce the melanin content in the final extract, it might however lead to low protein yield, most likely due to the loss of melanin-bound cellular proteins [335].

To further purify proteins from contaminants (e.g., salts, polysaccharides, fatty acids), protein precipitation in ammonium acetate in methanol is often the method of choice for highly melanized fungi, as it prevents protein re-solubilization problems frequently occurring when using TCA [330]. Pellet washes with organic solvents are additionally recommended to achieve a thorough clean-up. Additional strategies are sometimes necessary when dealing with species producing massive amounts of extracellular polysaccharides (EPS). Most of black fungi species present EPS matrices or capsules around the cell surface to enhance stress protection and to protect from infection [336]. If not removed during the extraction procedures, these polymers might hinder proteomic workflows and interfere with spectrophotometric methods [252], therefore high-speed centrifugation and/or filtration as well as sample dialysis before or after precipitation should be used [337].

*Collection of proteomics works*—The development of techniques for the separation of mycelial proteins and their identification by mass spectrometry, paved the way for the proteomic exploration of extremophile fungi by various criteria. In the black fungi group, which alone collects a large number of polyextremotolerant and polyextremophilic species, the first attempt to establish a workflow is however not older than one decade [330]. Not without extensive optimization, 2D-E or 2D-DIGE technology in combination with a wide spectrum of mass spectrometry techniques have ever since allowed to obtain little but crucial indications about metabolic strategies implemented by these organisms when dealing with sub-optimal living conditions. A qualitative and quantitative proteomic study conducted in rock-inhabiting black fungi—i.e., *Knufia perforans*, *Exophiala jeanselmei* and the Antarctic endemic *Friedmanniomyces endolithicus*—has brought to light how species with different ecology and distribution all react to temperatures far above their growth optimum by decreasing the number of expressed proteins. While this phenomenon suggests a reduced metabolism, the opposite trend was instead observed as a result of the exposure to temperatures far below the optimum [338]. A downregulation of the metabolism represented the typical response of extremophilic species of black fungi (i.e., *Cryomyces antarcticus*) also upon desiccation and the exposure to Mars-like conditions (Co2 and pressure) [339,340]. Conversely, mesophilic species such as *K. perforans* and *E. jeanselmei* tended to react to water loss by expressing additional proteins. It was therefore concluded that different black fungi species adopt similar strategies to cope with non-optimal environmental conditions, which involve the minimization of changes at the protein pattern level. The lack of a heat shock response (HSR) as part of a saving-energy mechanism promoting survival, indicates a stress response that differs quite dramatically from that found in mesophilic hyphomycetes [250], where a HSR is generally observed. Hence, it was suggested that a basic set of hardy temperature- and desiccation-resistant proteins is spread across the black fungi group, to flank morpho- and physiological characters in stress protection. The biological significance of these findings requires however future quantitative proteomic studies.

In a similar fashion, proteomics techniques have been applied to shed light on the mechanisms of adaptation to growth in alkaline conditions in the extremophile *Yarrowia lipolytica*, the only known ascomycete to grow on alkaline media and salt at near-saturating point [341]. 2D-E followed by MALDI-TOF-MS elucidated a key role for the stress protein Hsp12. The alkaline-inducible Hsp12, that provides a launch of emergency responses to a variety of stress allowing only a short-term survival in all studied yeasts under normal conditions, promotes rearranging and repairing of the membrane compartments under prolonged stress. Membrane potential homeostasis processes are also employed as cellular defense strategies to compete with lower environmental pH stimuli, as observed by [342]. Comparative analysis following 2D-E revealed a number of cellular processes employed as defense strategies to cope with acidic conditions, including metabolic flux shifting to α-ketoglutarate, known for its antioxidant role and of which *Y. lipolytica* appears to be a promising cell factory [341].

A gel-based proteomic approach was also deployed to investigate the adaptation to ionic stress in the extremophilic ascomicetous yeast *Debaryomyces hansenii* [343]. This species thrives in salty environments including polar waters and ice from Antarctic and Artic glaciers [344] and reportedly resorts to the intracellular accumulation of high levels of Na^+^ and K^+^ to achieve halotolerance [345]. Following cultivation in K^+^-free medium, Martinez et al. [343] observed striking changes in the proteome as a consequence of potassium starvation, with the majority of proteins found to be repressed. MALDI-TOF identification revealed how several of these proteins were involved in protein and amino acid synthesis, the Krebs cycle and the upper part of the glycolysis. The alteration of the global carbohydrate metabolism and the decrease in ATP production led the authors to conclude that an adaptive response is triggered by long-term potassium deprivation. Conversely, the upregulation of genes related to stress responses (i.e., HSP family of chaperones: Ssb1 and Hsp31), protein degradation (i.e., proteasome subunits and aminopeptidases) and sterols synthesis (i.e., Diphosphomevalonate decarboxylase Mvd1), was interpreted as a hallmark of a significant stress process. Further, in line with previous reports in *D. hansenii* and *Y. lipolytica* upon salt and pH stress respectively, changes in protein expression affecting membrane composition were observed under potassium starvation.

Sadaf et al. [346] used comparative proteomics to investigate the tolerance to ionic liquids (ILs) in the industrially relevant extremophile *Sporotrichum thermophile*, (syn. *Myceliophthora thermophile*). ILs are a class of solvents with applications in the area of biomass processing, which exert toxic effect towards microorganisms and most of saccharifying enzymes (e.g., cellulases and xylanases). Following a 24-h exposure to various concentrations of ILs supplied in the cultivation medium, 2D-E and LC-MS-ToF revealed distinct changes in the IL-treated gels. Global proteome analysis showed an ILs-induced oxidative stress that triggered the metabolic regulation of many significant pathways as well as the expression of HSP70 and anti-oxidative enzymes (i.e., catalase/peroxidase). Upregulation of glycolysis, pentose phosphate pathway and ATP synthesis was detected under ILs-stress, alongside the downregulation of protein biosynthesis and TCA cycle, which was also observed in Paracoccidioides yeast cells coping with H_2_O_2_-induced oxidative stress. Based on such metabolic regulation, the authors proposed that ILs can be used to manipulate protein behavior.

In an effort to identify proteins associated with survival to deep-sea conditions, shotgun proteomics was carried out in isolates from deep-sea sediment [331]. In this study, four filamentous fungi—*Aspergillus terreus*, *Aspergillus flavus*, *Aspergillus sydowii* and *Penicillium* sp.—were incubated at 4 °C under various hydrostatic pressures. LC-MS-quadrupole time of flight (QToF) analysis resulted in the detection of proteins commonly up- and downregulated across all elevated pressure conditions when compared to the control, in both mycelial and conidial samples. Interestingly, house-keeping proteins had higher fold-change regulation than stress proteins, with proteins involved in glycolysis, pentose-phosphate-pathway, Krebs cycle, electron transport, signaling, cell-wall generation, cell division and cell growth being mostly downregulated. The downregulation of essential proteins was suggested by the authors to be at the base of the slow growth recorded in deep-sea conditions, which would be crucial to enable survival under stressful conditions.

Romsdahl et al. [347] applied shotgun proteomics to the molecular characterization of a novel strain of *Aspergillus niger* isolated from the International Space Station (ISS). Tandem Mass Tag (TMT) labelling coupled with reverse phase LC-MS/MS was used to get an insight into the adaptive mechanisms to space travel conditions at the proteome level. Protein functional and pathway analysis confirmed that differential abundance affected especially proteins involved with starvation and stress response and nutrient acquisition. As speculated by the authors, the upregulation of starvation-induced enzymes (e.g., glycoside hydrolases) may point to the adaptation to the oligotrophic ISS environment [348], whereas the increased expression of catalase and kinases is to be connected with the oxidative stress response, triggered by the enhanced irradiation environment of the ISS [349,350]. This is in line with the increased abundance of the polyketide synthase AlbA, a key enzyme involved in the synthesis of DHN-melanin, reportedly found to be upregulated also in fungi isolated from high-radiation environments and exhibiting increased melanin production [351]. Altogether, this study revealed the existence of a distinct strain of *A. niger* onboard the ISS exhibiting higher melanin content and differential growth pattern and protein phenotype than its Earth counterparts.

Blachowicz et al. [352] applied TMT-LC-MS/MS to detect alterations in protein expression in extremophilic filamentous fungi, with a focus on survival to simulated Mars-like conditions (SMC). This study showed how the exposure to SMC caused proteome alterations in both the model organisms: the ISS-isolate *Aspergillus fumigatus* and *Cladosporium cladosporioides*, the latter of which was isolated from the Chernobyl nuclear-power-plant, when compared to unexposed counterparts. Ribosome biogenesis, translation and carbohydrate metabolic processes were the mostly affected pathways in both species. The SMC-induced increased abundance of the ribosomal protein Rpl17, indicated as crucial for survival in *A. fumigatus* in previous reports [353], was suggested by the authors to modulate the fungus response to harsh conditions. Similarly, a number of enzymes involved in carbohydrate metabolism and energy conversion and supporting growth on alternative C2 carbon sources—e.g., the AcuD and AcuE lyases from the glyoxylate cycle—were upregulated. Based on the upregulation of enzymes involved in chitin recognition and degradation, the recourse to chitin debris as alternative carbon source was speculated in *C. cladosporioides*. This further suggests alterations in carbohydrate metabolism to be an adaptive response to SMC.

#### 4.2.2. Secretomics

Secretomics of fungal extremophilic and extremotolerant species has recently gained momentum in view of the increasing demand of new natural sources of bioactive compounds by the biotech, pharmaceutical and cosmetic industry [354,355]. Proteomics tools are therefore being increasingly applied to the identification of microbial enzymes of biotechnological value and their structural and functional characterization [356,357,358].

*Methodological strategies in sample preparation*—Major difficulties encountered while attempting to the isolation of secreted proteins from extremophiles are related to lipids and polyphenolic compounds, largely synthesized in species coping with severe abiotic stresses. Different methods for sample clean-up are generally used to aid the removal of contaminants and reduce their interference with downstream procedures for protein quantitation, separation and analysis (Figure 4B). The method of choice is species-specific and requires a case-by case assessment, especially since standard methodologies might not be effective, and optimization might be necessary. In addition to protein precipitation, filter-aided sample preparation (FASP) methods for protein in-solution digestion prior to MS, was recently reported to successfully eliminate melanin from heavily pigmented secretomes in the black fungus *Knufia chersonesos (syn petricola.)* without dramatic downturns of the protein yield [24]. This method proved particular effectiveness in separation of melanin and EPS, retained on top of the membrane filter, from fungal peptides which were eluted in a colorless buffer.

*Collection of secretomics works*—Although initial researches on secretome had focused on few of the representative fungal genera belonging to Ascomycetes [359], the list of species analyzed by this comprehensive approach has ever since slowly expandd to less widespread extremotolerant and extremophilic species. Yet, to date the number of secretomics reports on industrially relevant strains and fungal pathogens still outnumbers the studies conducted on extremophiles.

Arfi et al. [360] sought to characterize salt-adapted lignocellulolytic enzymes in the halotolerant mangrove fungus *Pestalotiopsis* sp., whose mechanisms for biomass degradation were very poorly known. Secretomes from cultures grown on the fungus natural lignocellulosic substrate (i.e., mangrove wood chips) with or without salt, were analyzed by LC-MS/MS and identified by mass-matching against an in-house database derived from the transcriptome of *Pestalotiopsis* sp. NCi6. Differences were observed between the sets of proteins expressed at each condition and a lower enzymatic diversity was found in the saline secretome. Proteomic analyses showed that salt affects lignocellulolytic enzyme composition, this resulting in an increase in xylanases and cellulases and a decrease in oxidases. Similarly, enhanced hemicellulolytic and cellulolytic activities were observed alongside a reduction in lignin breakdown. Further, the adaptation to saline condition was alsoreflected by the presence of multiple proteins of the same enzyme family (e.g., glycoside hydrolase 43), which, the authors speculated, would represent isoenzymes specifically adapted to salinity. Although further studies are required to thoroughly explore the salt-tolerant secretome repertoire, these findings underline the biotechnological potential of *Pestalotiopsis* species.

A more recent study of lignocellulolytic enzymes reports on the secretome analysis of the thermophilic fungus *Malbranchea cinnamomea*, naturally found in composting soil and known for the production of the quinone antibiotic malbranicin [361]. LC-MS/MS orbitrap showed a rich asset of enzymes involved in classical and oxidative cellulolytic mechanisms, a number of which were catalytically efficient proteins capable to enhancing the hydrolysis of alkali treated carrot grass (ATCG), a widespread obnoxious weed. Furthermore, metal-dependent enzymes (i.e., protein hydrolase and di-peptidase)—modulated by divalent metal ion (Mn^2+^/Cu^2+^)—were also observed and found to mediate the catalysis of selected enzyme fractions for achieving improved hydrolysis. A secretome screening towards thermostable enzymes was also performed for the thermophilic mold *Mycothermus thermophilus* (syn. *Humicola insolens*), known to be associated with decaying lignocellulolytic materials [362]. Mass spectrometry analysis (Q-TOF LC/MS) of cultures on different cultivation media, revealed the production of a diverse set of glycosyl hydrolases—e.g., cellobiohydrolase I, β glucosidase, endoglucanase, xylanase—and auxiliary proteins. This suggests the strain as an important source of lignocellulolytic CAZymes, a large class of enzymes which build and breakdown the complex carbohydrates of the cell [355]. The activity of some of the cellulases was additionally shown on alkali treated rice straw and bagasse, which were thereby efficiently hydrolyzed into fermentable sugars. Zymography and shotgun proteomics (LC-MS/MS) were also applied to profile the secretome of the thermophilic fungus *Thermomyces lanuginosus* and revealed the production of novel enzymes important to various industrial processes (e.g., amylases, lipases, aminopeptidases, etc). In particular, the data provided insight into the fungus ability to utilize lignocellulosic material despite the lack of cellulases, which relies on the hyper-production of a thermostable, pH tolerant xylanase [363].

Further secretomics investigations of enzymes of biotechnological interest were carried out in the coprophilous ascomycetous fungus *Doratomyces stemonitis* [364]. Although not strictly an extremophile, the fungus survival largely depends on the ability to utilize highly recalcitrant and indigestible parts of the plant, found as waste product in herbivore feces. Both gel-based (including zymography) and shotgun techniques were applied by the authors to the identification of enzymes involved in the degradation of cellulose, hemicellulose, pectin and lignin. Following fungal growth in a hydrolase-inducing liquid medium, a combination of MALDI-TOF/TOF MS/MS and/or Q-TOF LC-MS/MS revealed a secretome dominated by cellobiohydrolase of the glycosyl hydrolase family 7 (GH7), endoglucanases (GH5 and GH74) and β-glucosidases (GH3), and where the endo-1,4-β-xylanase of the GH10 family was the most abundantly identified protein. These main classes of enzymes involved in cellulose degradation are commonly identified in the secretomes of saprophytic fungi [365], however a higher degree of specialization for pectin degradation was observed in the secretome of *D. stemonitis*.

In a similar attempt to explore novel enzymes for biodegradation, Tesei et al. [366] have investigated secretome alterations in the rock black fungus *Knufia chersonesos* (syn. *petricola*) in response to the synthetic copolyester polybutylene adipate terephthalate (PBAT). Label-free shotgun quantitative proteomics unveiled a total of 37 esterolytic and lipolytic enzymes in both *K. chersonesos* and a non-melanized mutant of the same species spontaneously originated under laboratory conditions. Out of 37 enzymes, ninewere constitutively expressed and sevenwere instead detected as upregulated or induced by PBAT in minimal medium cultures, where the polyester represented the sole carbon source. Protein functional analysis and structure prediction proved similarity of some of these enzymes with microbial polyesterases of known biotechnological application—e.g., MHETase from *Ideonella sakaiensis*, CalA from *Candida antarctica* and feruloyl esterase b from *Aspergillus oryzae*—which is suggestive of their potential role in PBAT degradation. In line with these findings, PBAT complete hydrolysis was recorded by spectrophotometric determination at all cultivation conditions, but at higher levels in minimal medium. Differences in the degradation ability were not observed between wild type and mutant strain, however the mutant secretome showed lower diversity. Hence, cell wall melanisation does not seem to play a role in the strain predisposition to polyester degradation. The hydrolytic ability of single enzymes shall be validated with cloning and expression studies. Nonetheless, these results remark the biotechnological significance of extremotolerant RIF and their aptitude to resort to alternative carbon sources while also suggesting the interconnection between rock lifestyle and biodegradation [367].

#### 4.2.3. Membrane and Cell Wall Proteomics

The sheltering function of the fungal cell wall from environmental stress makes the investigation of the associated proteins very appealing. Nonetheless, mostly due to the complexity of protein extraction, membrane and cell-wall proteomics of extremophilic and extremotolerant fungiis still in its infancy, which reflects the extremely low number of studies. If the characterization of organelle proteins in species from the extremes is yet to be accomplished, optimization work is also required to adapt pre-existing procedures to achieving efficient removal of melanin, lipids and polysaccharides. Enzymatic methods like cell wall digestion and protoplast formation may represent in this regard a valuable option, as long as they do not affect the physiology of the fungus (Figure 4C). In black fungi, a protoplast-based system for genetic transformation was recently established and could possibly serve as a starting point in proteomic applications to aid the isolation of cell wall and membrane proteins [368].

A quite recent report on fungal sub-proteomic analysis used 2D-E and label-free shotgun proteomics to detect differential expression of *Penicillium oxalicum* cytosolic and membrane proteins in response to the polycyclic aromatic hydrocarbon (PAH) anthracene [369]. As the strain, isolated from a hydrocarbon-polluted environment, is involved in the transformation of various PAHs, the authors aimed to identify the process by which this fungus metabolizes anthracene as well as to detect molecular disturbances in the sub-proteome. Following culture exposures to anthracene for 48 h, overexpression of proteins related to stress response was detected in both the cytosolic and the microsomal fractions. Similarly, different antioxidant enzymes were found in both fractions. Anthracene was found to enhance the expression of several proteins involved in the metabolism of xenobiotics such as cytochrome p450s (CYPs) and a number of epoxide hydrolases and transferases enzymes known to contributing to the PAH biodegradation pathway. These results reinforce the involvement of the fungus and of specific classes of proteins in anthracene degradation and remarks the need of a deeper knowledge on fungal proteomics for the application of the appropriate microorganisms in biodegradation processes.

### 4.3. Proteomics in Lichens

Though proteomic approaches have been shown to have a great potential to study non-model species lacking genetic information, few studies have utilized proteomics to investigate lichens or lichen symbionts. This mostly depends on the struggles in the extraction of lichen-forming fungal proteins, which is largely hindered by phenols, quinones, proteases and other components released following cell disruption [370]. Furthermore, as also in the case of extremophilic fungi, the difficulty of breaking the cell-wall hinders the achievement of reasonable concentration of proteins. Protein profiling and differential analysis works have hitherto contributed to get an insight into the lichens’ mechanisms of tolerance under stress conditions, however the proteome of lichens is far from being completely elucidated.

*Methodological strategies in sample preparation*—Lichen proteins isolation is achieved resorting to protocols initially developed for protein extraction from recalcitrant samples such as plant tissues [371]. Mechanical disruption of the biomass under liquid nitrogen is very often used in combination with homogenization buffers containing proteases inhibitors and components like polyvinylpolypyrrolidone (PVPP), that can effectively reduce the influence of phenolics and derivatives in the extract. Ascorbic acid can be applied to the ground material before addition of the buffer solution to aid the removal of quinones and thus reduce the pigmentation of the extract. In order to remove extracellular acetone-soluble phenolics, the lichen thalli are at occurrence washed multiple times with acetone prior to cell lysis and then air dried [370]. Extraction might require extensive stirring of the sample at low temperature, followed by protein precipitation steps with TCA and acetone. Pellet washes in methanol or acetone are required to improve removal of interfering compounds [372]. Along with the sample preparation, an additional aspect to consider is the co-occurrence of at least two symbiotic organisms, which makes it essential to apply bioinformatics methods to discriminate and assign proteins.

*Collection of proteomics works*—First proteomics reports in lichens date back to three decades ago, when studies on *Xanthoria parietina* were performed aiming at the detection of proteins involved in the lichen-algae interplay. 1D-E was employed for the detection of algal-binding proteins (ABP) and binding assays allowed to substantiate the interaction between ABP and phycobiont cells, which the authors speculated to be of polysaccharide nature [373]. A number of electrophoretic studies have been made with taxonomic objectives and elucidated the enzymatic variation between or within populations or morphotypes. The work of Hageman et al. [374] on *Umbilicaria* gave a first insight into inter and intraspecific similarity at the proteome level showing how the similarity between conspecific lichens with different morphology belonging to the same population was greater than between specimens with similar morphology belonging to different populations. Kershaw et al. [375] characterized the potential range of enzyme polymorphism in two contrasting morphotypes of *Cladonia stellaris* from sun and shade locations and demonstrated a good level of isozyme homogeneity between the two morphotypes studied. Seasonal variation in isoenzymes was observed in specimens of three subspecies from different populations which had different enzymatic and protein characteristics [376].

The fast development of a wide variety of proteomics techniques has paved the way for more complex studies. Later investigations have increasingly focused on proteins involvement in stress survival and on biomarkers of stress, also in view of the common utilization of lichens as biomonitors of metal pollutants present in the environments. Rustichelli et al. [372], employed a 2D-E, MALDI-TOF-MS approach to dissect cadmium (Cd) stress response in the foliose macrolichen *Physcia adscendens*, known for its high adaptation to highly polluted environments [377]. Protein differential analysis revealed the increased expression of glutathione S-transferase and heat-shock proteins in relation to in vitro Cd treatment, with HSPs possibly acting as direct chelators of intracellular Cd to reduce the potentially deleterious effects of free Cd ions. By contrast, ABC transporters were under-expressed after short-term exposure (i.e., 6–18 h), but in some cases induced after longer exposure to Cd. The cytochrome P450, enzyme involved in the catabolism of a plethora of xenobiotics and in protection against oxidative stress, appeared to have a variable expression pattern over time, with higher levels reached in response to short Cd treatments. It was instead inhibited in condition of a long exposure to the metal. A similar study on the adaptive response to metals investigated the changes of the lichen proteome during exposure to constant concentrations of mercury [378]. A proteomic approach (2D-E in combination with LC-MS/MS) applied to the epiphytic *Evernia prunastri* (L.) Ach., species widely utilized as bioindicator of metal effects, revealed that most of changes involved proteins of the photosynthetic pathway. This is in line with the fact that the photosynthetic pathway is a usual target of metal contaminants and was substantiated by observed lower concentration of photosynthetic pigments and alterations of the plastid ultrastructure. Specifically, the chloroplast ATP synthase subunit beta increased in concentration following Hg exposure but rapidly dropped down below basal levels, while the content of mitochondrial ATP synthase increases progressively indicating that lichens might counterbalance the decrease in chloroplast ATP production by increasing the content of mitochondrial ATP synthase. The Rubisco large subunit (form I and II) accumulated in response to Hg exposure, which, as speculated by the authors, could be connected to the decreased content of the chloroplast photosystem I reaction center subunit II and of NADPH. Furthermore, the level of Hsp70 increased considerably during treatment, suggesting an undergoing protective response to Hg exposure. These data supported the idea that adaptation to Hg in lichens is likely to involve changes in the photosynthesis-based metabolism coupled with detoxification and stress response mechanisms.

Proteomics investigations also aimed at deepening the understanding of the impact of symbiosis on the lichens’ resilience to environmental stress. The pioneer study of Schneider et al. [379] characterized the proteome of the lung lichen *Lobaria pulmonaria* and found functions specifically associated either to the fungal or algal proteins but also to prokaryotic proteins, whose number was as high as the number of normalized spectral counts of the green algal photobiont. A major proportion of fungal and bacterial proteins, which constituted 84% of all proteins identified by semi-quantitative label-free proteomics, were found to be involved in PTMs and protein turnover whereas the proteome of the photobiont was found to be mostly associated with energy and carbohydrate metabolism. These results thereby confirmed the symbiotic function of the photobiont and the importance and multiple functions of the bacterial partner in the symbiotic ecosystem. More recently, the microbiome associate to *L. pulmonaria* was screened for functions encoded in genomes and their expression at the protein level was verified using an integrated metagenomics and metaproteomics approach [380]. The results of this study provided strong evidence that bacterial species contribute several aspects to the symbiotic system including nutrient provision, pathogen and stress defense, biosynthesis of vitamins and hormones, detoxification processes and degradation of older lichen thallus parts. Additionally, the high prevalence of bacterial nitrogen fixation was confirmed with—omics data and quantitative RT-PCR. A new metaproteomics analysis of *L. pulmonaria* was carried out more recently by Eymann et al. [381] in an attempt to assess structure and functionality of the entire lung lichen collected from different sites. Based on the observed protein pool, a lichenicolous fungus and a complex prokaryotic community could be detected in addition to the already known green algae and ascomycetous fungus and various partner-specific proteins could be assigned to the different symbionts. The observed functional diversification—e.g., algal proteins involved in photosynthesis, fungal proteins involved in vesicle transport, cyanobacterial nitrogenase and GOGAT involved in nitrogen-fixation and bacterial enzymes involved in methanol/C1- compounds metabolism as well as CO-detoxification—was interpreted by the authors as a convenient strategy to support the longevity of *L. pulmonaria* under certain ecological conditions.

Munzi et al. [382] sought to expand the understanding of nitrogen stress effects on the proteome of *Cladonia portentosa*, reportedly able to cope with increased N availability, and found that different N forms affected metabolic pathways in the mycobiont and the photobiont differently. Changes in protein expression were mostly detected in the fungal partner in response to NO_3_^−^ and NH_4_^+^, which impacted the energetic metabolism and protein synthesis, respectively. Conversely, the photobiont only showed overexpression of enzymes responsible for energy production and photosynthesis—the latter of which is commonly observed as effect of metal contamination (e.g., subunit beta of the ATP synthase and fructose–bisphosphate aldolase)—upon different doses of both N forms. These proteomic data thereby provided evidence at the molecular level that N tolerance is possible thanks to the energy supply provided by the photobiont and used by the mycobiont to maintain functionality in the presence of NO3.

### 4.4. Use of Proteomics for Species Identification

Fungal investigations by means of proteomics are not limited to the exploration of the protein pool but also involve the identification of microorganisms in culture. The possibility to perform mass spectrometry on intact cells using MALDI-ToF-MS has in fact paved the way for several microbiology applications of proteomics, especially since advances in informatics allowed the connection of microbial MS databases to automated computer-based analytics. Such progresses led to the standardization of the method and to the regulatory approval of MALDI-ToF-MS systems for routine identification of bacteria, yeasts, dimorphic and filamentous fungi in medical centers and clinical microbiology laboratories worldwide [383].

The discrimination among microorganisms is based on the highly specific peptide profiling of the most abundant proteins, like ribosomal proteins, and has proven relatively low costs, speed of analysis (turnaround time of approximately 10 min) and great accuracy in several cases [384]. The resultant mass spectrum represents a signature profile of individual organisms, where peaks can be specifically assigned to clusters, genera or species, depending on relatedness of the test organism to closely related ones, included in a database of reference spectra. As for any identification system, the availability of precisely compiled and validated proteomic databases is critical to achieve accurate results and to prevent misidentifications, quite commonly observed especially in clinical mould isolates [385]. However, MALDI–ToF-MS has in due course become a valid alternative to extensively employed identification techniques using ribosomal DNA (rDNA) genes sequencing, especially for analyses involving a large number of samples. Hence, proteome phenotype profiling has been performed on many fungal genera such as *Aspergillus*, *Fusarium*, *Penicillum* or *Trichoderma*, including common and uncommon clinically relevant isolates [386,387,388,389].

MS-based microbial diagnosis is especially relevant in species where definitive identification through classical morphological methods is hampered by the lack of distinct reproductive structures, like in black fungi and black yeasts. Nonetheless, the number of reports on MALDI-based identification of black fungi is still little mostly due to the notable limitation of historical fungal database [390,391]. Additionally, the presence of pigments in the samples may lead to signal suppression and thereby result in poor fingerprint mass spectra [392]. Recently Paul et al. [383], attempted to standardize sample preparation and their work culminated in the creation of an in-house database for the rapid identification of melanized fungal isolates. As such, MALDI-TOF-MS is likely to rapidly evolve in the near future due to systematic and continuing library updates and to extend its application to the rapid and accurate detection of a higher number of species, including emerging clinically important fungi and melanotic strains.

### 4.5. Bioinformatics Tools for Fungal Protein Analysis

The progress in proteomics technologies and the consequent increase in MS resolution that occurred during the last decade, has set the stage for global studies of the proteome and led to the generation of large and heterogeneous data sets. This, in turn, has posed analytical challenges hitherto unseen by protein researchers. If the development of computational proteomics—statistical and machine learning algorithms e.g., the open-source proteomics software package MaxQuant/Perseus (https://www.maxquant.org/) and Skyline (https://www.skyline.ms)—became necessary to facilitate the extraction of protein qualitative and quantitative information from MS data, the mapping of complex proteomics data to biological processes has become impossible by manual means, which made the recourse to bioinformatics algorithms essential to data mining.

The application of high-throughput-omics approaches such as next generation sequencing and transcriptomics has paved the way for new sub-discipline of bioinformatics such as proteogenomics, whose aim is to harness MS-based proteomics data sets in conjunction with DNA sequence data sets for large scale genome and proteome annotation [393]. Similarly, the combination of protein data sets with transcriptomics has allowed to spot correlations between mRNA expression levels and protein abundances. Thus, peptide libraries have been used to find novel transcripts and to refine gene models, this leading to improved genome and proteome annotations, augmented databases and thereby an increased number of identifications [394].

Though the achievement of a thorough understanding of protein functions and interactions is a crucial aspect of any proteomics workflow that aims to elucidate an organism cell physiology, it is not free from challenges especially when dealing with non-model organisms. Furthermore, if the genome sequences of several clinically relevant or phytopathogenic fungi have been made available at early stages, the completion of annotation of genomic data in less known species e.g., extremomophiles has proceeded at a much lower speed. Such delay has largely hindered the functional exploration of these organisms.

Protein functional analysis is to some extent possible even when RNA-seq-based genome annotations are unavailable, however it entails the bioinformatic-assisted generation of databases of predicted proteins. AUGUSTUS is to date the best-known tool for the gene prediction of non-model and emerging model organisms, as it enables the “ab initio” translation of genome sequences based on annotated genomes from closely related species, used as training databases [395]. Protein identifications and functional insights are obtained searching for sequence homologs using HMMER (http://hmmer.org/) together with profile databases such as PFAM (http://pfam.xfam.org/) or Interpro (http://www.ebi.ac.uk/interpro/). Both PFAM and Interpro provide functional information by predicting protein domains and critical functional sites, however PFAM generates higher-level grouping of related entries, known as CLANS, while Interpro classifies proteins into families. In species whose complete genome sequences have been assembled and annotated, the achievement of protein identifications is way more straight-forward.

Bioinformatics analysis generally involves integration of proteome data with annotation databases such as Gene Ontology (GO) and pathway databases (KEGG) to determine overrepresented biological processes and molecular functions. Functional insights into the data can be obtained resorting to readily available free online resources for data mining like the very comprehensive database of protein sequence and functional information UniProt (http://www.uniprot.org)—that includes manual annotation of fungi-specific proteins and protein families (Fungal Protein Annotation Project; https://www.uniprot.org/program/Fungi/)—the PRoteomic IDEntifications database (PRIDE, www.ebi.ac.uk/pride/), the Database for Annotation, Visualization and Integrated Discovery (DAVID; https://david.ncifcrf.gov/), the Protein Analysis Through Evolutionary Relationships (PANTHER; http://www.pantherdb.org/about.jsp) and the integrated functional genomics database FungiDB (http://fungidb.org/fungidb/), specifically created for fungi and oomycetes [396]. Additional tools—e.g., the web server REVIGO—can be used to summarize long and unintelligible lists of GO terms associated to the groups of proteins with changed abundance, by clustering semantically close terms [397]. As for non-free alternatives, an interesting resource is represented by the bioinformatics software OmicsBox, which includes a functional analysis module based on the Blast2GO annotation methodology as well as a module for enrichment and statistical analysis (https://www.biobam.com). Although it requires some more programming experience, the R statistical platform is also widely employed as it offers broader flexibility in the analysis (https://www.r-project.org/).

Bioinformatics tools can also allow predictions of localization for a large number and a great variety of proteins, solely based on the amino acidic sequence. This aspect holds particular importance in all those species where, as described earlier, the characterization of the subcellular proteome can be especially cumbersome. Tools for the localization of intra- and extracellular proteins in fungi have been developed and include WoLF PSORT, MultiLoc2, SherLoc2, MSLoc-DT, SCLpred and BUSCA, among others [398,399,400,401,402,403]. Advanced platforms e.g., SignalP 5.0 [404] (http://www.cbs.dtu.dk/services/SignalP/) and Phobius [405] (http://phobius.sbc.su.se/cgi-bin/predict.pl), enable analyzing proteins for sequence features like signal peptides and transmembrane regions. The Fungal Secretome KnowledgeBase (FunSecKB and FunSecKB2; http://bioinformatics.ysu.edu/secretomes/fungi.php) [406] and the Fungal Secretome Database (FSD; http://fsd.snu.ac.kr/) have been established to collect extracellular proteins from all available fungal protein data in the NCBI RefSeq database. The FSD, at present time the most accurate platform for putative secretory proteins, uses different databases like SigCleave, SIgPred and RPSP to screen those proteins not considered positive by SignalIP [34].

Further information about proteins, from molecular weight and isoelectric point to prediction of *N*-glycosilation sites and much more, can be retrieved resorting to free online tools such as those comprehensively listed on the ExPASy Bioinformatics Resource Portal (https://www.expasy.org/proteomics). This can elucidate features of studied samples while also helping to ascertain and to correct experimental or MS-identification related sampling biases in proteome libraries [393]. The Fungal Genome (http://fungalgenomes.org/), Fungal-Blast (https://www.yeastgenome.org/blast-fungal), MycoBank (http://www.mycobank.org/), the Sanger Institute Fungal Sequencing (http://sanger.ac.uk/Projects/Fungi/) and the Q Bank (http://www.q-bank.eu/fungi/) for species that are of relevance to mycological phytopathology, are instead examples of fungal genome and nucleotide sequences databases.

The technological progresses which have allowed to obtain accurate global qualitative and quantitative proteome data by MS have also encouraged studies of the proteome dynamic networks and post-translational modifications. Proteomics-based methods and bioinformatics-based approaches have in this respect largely replaced genetic and biochemical methods of former application in the exploration of protein-protein interaction (PPI) and PTMs [407,408]. Both PPI and PTMs have uncountable effects on cell metabolism and physiology, also being key process regulators of extremotolerance and stress resistance. A number of bioinformatics tools used for protein functional analysis include the PPI annotation category (e.g., DAVID, KEGG, ExPaSy etc.). Several databases are also available for studying PPI networks, like the Database of Interacting Proteins (DIP; https://dip.doe-mbi.ucla.edu/dip/Main.cgi), the Molecular Interaction Database (MINT) from UniProt (https://mint.bio.uniroma2.it/), and the IntAct molecular interaction study tool (https://www.ebi.ac.uk/intact/). Many bioinformatics tools have been developed to support the analysis of PTMs by mass spectrometry, from prediction to PTM site assignment. The list of public databases available for searching for PTM annotation include type-specific databases such as Phospho.ELM (http://phospho.elm.eu.org/) and others which cover more modification types such as Uniprot, PHOSIDA (http://141.61.102.18/phosida/index.aspx) and PTMcode (https://ptmcode.embl.de/). Some only include experimentally verified PTMs such as Phospho.ELM, PhosphoSitePlus (https://www.phosphosite.org/homeAction.action) PhosphoGrid and UniCarbKB, some include known and predicted functional annotations between PTMs such as iPTMnet.

The relatively large body of bioinformatics methods mentioned above, will in the future increasingly aid the analyses of protein sequences, structures, modifications and networks, thereby having the potential to revolutionize our understanding of biology, disease, stress resistance and of the dynamics of the epiproteome.

## 5. Conclusions

In this review we presented an overview of the major advances in genomics, including phylogenomics, and proteomics of euascomycetes (Ascomycota), in particular reporting on examples selected from plant and animal opportunistic and pathogenic, extremophilic/polyextremotolerant filamentous and yeast-like micromycetes, as well as lichenized fungi. We also included some notions and concepts on methodological strategies and bioinformatics tools applied for sample preparations, genome and proteome sequence data analyses, respectively. Notwithstanding the huge amount of studies conducted on eumycetes, this contribution aimed at being as more comprehensive as possible and we hope to offer with it a roundup of the most recent—omics studies and the applied approaches. Though the achievement of a thorough understanding of protein functions and interactions is a crucial aspect of any proteomics workflow in order to elucidate an organism cell physiology, it is not free from challenges especially when dealing with non-model organisms. Furthermore, if the genome sequences of several clinically relevant or phytopathogenic fungi have been made available at early stages, the completion of annotation of genomic data in less known species e.g., extremomophiles has proceeded at a much lower speed. Such delay has largely hindered the functional exploration of these organisms so far.

The relatively large body of new results and bioinformatics methods mentioned above, will in the future increasingly aid the analyses of geniome and protein sequences, structures, modifications and networks, thereby having the potential to revolutionize our understanding of biology, disease, stress resistance and of the dynamics of fungal life styles in general.

## Appendix A

**Table A1:** List summarizing the reviewed lichen genomics works.

Lichen Species	Lichen Material	Applied Approach(s)	Remarks	References
*Cetradonia linearis*	whole thallus, nuclear genome	whole-genome shotgun Illumina sequencing; SNP calling	sequencing of reference genome and population genomic analysis	[116]
*Cladonia grayi*	cultured mycobiont, nuclear genome	Illumina genome sequencing; transcriptome sequencing; analyses of gene families;	identification of genes/proteins of potential symbiotic relevance	[123]
*Arthonia radiata*	cultured mycobiont, nuclear genome	Illumina genome and transcriptome sequencing; assamply with overlapping paired-end (PE) and mate pair (MP) libraries	sequencing and assembly of genome and transcriptom	[409]
*C. apodocarpa*, *C. caroliniana*, *C. furcata*, *C. leporina*, *C. petrophila*, *C. peziziformis*, *C. robbinsii*, *C. stipitata*, *and C. subtenuis*	whole thallus, mitochondrial genome	Illumina sequencing; phylogenetic analysis (Bayesian MCMC)	analysing genome size, protein coding gene content, intron-encoded retrotransposable elements, Bayesian analyses to assess efficacy of five loci	[125]
*Lasallia hispanica*	cultured mycobiont, nuclear genome	Illumina sequencing	genome asembly and annotaion, comparison with the closest related species	[131]
*Usnea halei*, *U. mutabilis*, *U. subfusca*, *U. subgracilis*, *and U. subscabrosa*	whole thallus, mitochondrial genome	Illumina sequencing	genome asembly and annotaion, phylogenetic infeence with the closest related species	[127]
*Lasallia pustulata*	whole thallus, metagenome	whole-genome shotgun (metagenome skimming), Illumina sequencing	testing the best genome ssembling method	[131,140]
*Usnea antarctica*, *U. aurantiacoatra*	whole thallus, metagenome	RADseq	population genomics and species distinction	[136]
*Rhizoplaca haydenii*, *R. idahoensis*, *R. melanophthalma*, *R. novomexicana*, *R. occulta*, *R. parilis*, *R. polymorpha*, *R. porteri*, *R. shushanii*	whole thallus, metagenome	RADseq	phylogenomics	[135]
*Cladonia rangiferina*	cultured mycobiont, nuclear genome	Sanger EST sequencing data	characterization of transcriptome	[124]
*273 lichen species*	whole thallus, nuclear genome	whole-genome shotgun Illumina sequencing	comparison of WGS vs. amplicon sequencing efficiency; species identification for ecological pourposes	[139]
*Rhizoplaca melanophthalma (species complex)*	whole thallus and cultured mycobiont, nuclear and mitochondrial genomes	genome Illumina sequencing	phylogenomic; to infer evolutionary relationships and potential patterns of introgression	[133]
*Rhizoplaca melanophthalma (species complex)*	whole thallus and cultured mycobiont, nuclear genomes	genome Illumina sequencing	phylogenomic	[134]
*Alectoria sarmentosa*	whole thallus, metagenome	genome Illumina sequencing	sequencing of draft genome	[141]
*Acarospora strigata*, *Arthonia rubrocinta*, *Dibaies baeomyces*, *Endocarpon pallidum*, *Graphis scripta*, *Leptogium austroamericanum*, *Peltula cylindrica*, *Physcia* cf. *stellaris*	whole thallus and cultured mycobiont, nuclear genomes	whole-genome shotgun, Illumina sequencing	to study the evolution of the ammonium transporter/ammonia permease gene family	[118]
*Evernia prunastri, Pseudevernia furfuracea*	whole thallus and cultured mycobiont, nuclear genomes	whole-genome shotgun, metagenome skimming, Illumina sequencing	to test accuracy and completeness of assemblies based on metagenomic sequences and comparison with assemblies based on pure cultured strains	[115]
*Caloplaca flavorubescens*	cultured mycobiont, nuclear genome	whole-genome shotgun, Illumina sequencing	sequencing of draft genome	[119]
*Cladonia macilenta*	cultured mycobiont, nuclear genome	whole-genome shotgun, Illumina sequencing	sequencing of draft genome	[120]
*Umbilicaria muehlenbergii*	cultured mycobiont, nuclear genome	whole-genome shotgun, Illumina sequencing	sequencing of draft genome	[121]
*Cladonia metacorallifera*	cultured mycobiont, nuclear genome	whole-genome shotgun, Illumina sequencing	sequencing of draft genome	[122]
*41 lichen species*	whole thallus, nuclear genome	whole-genome shotgun Illumina sequencing	to characterize the MAT locus for heterothallism or homothallism	[132]
*58 lichen species*	whole thallus, mitochondrial genome	whole-genome shotgun Illumina sequencing	to analyze mitochondrial genome evolutionby loss of introns	[142]
*Ramalina intermedia*	whole thallus, nuclear genome	whole-genome shotgun Illumina sequencing	draft genome sequence	[128]
*Endocarpon pusillum*	cultured mycobiont, nuclear genome	whole-genome shotgun Illumina sequencing	genome sequencing and characterization for symbiotic traits	[130]
*Physcia stellaris*	cultured mycobiont, nuclear genome	SpotON R9.4.1 FLO-MIN106 flowcell and Illumina sequencing	draft genome sequence	[129]
*Peltigera malacea*, *P. membranacea*	whole thallus, mitochondrial genome	Illumina sequencing	comparative analysis and phylogenetic infeence	[126]

## Appendix B

**Table A2:** List summarizing the reviewed proteomics works.

Fungus	Fungal Material	Experimental Workflow	Number of Identified Proteins	Remarks	References
*Aspergillus flavus*	Conidia, whole cell proteome	LC-MS-QToF	Not indicated	Identification of proteins expressed under simulated deep-sea condition	[331]
*Aspergillus fumigatus*	Mycelium, whole cell proteome	iTRAQ LC-MS/MS	471	Quantitative proteomics of the fungus response to caspofungin to determine potential biomarkers of drug action	[261]
	Conidia	*SWATH-MS*	712	Time course evaluation of the conidial proteome	[265]
	Secretome	LC-MS/MS	484	Secretome profiling	[302]
	Cell wall proteome	UHPLC-MS^E^	367	Detection of protein targets for broad-spectrum mycosis vaccines	[325]
		LC-MS/MS	148	Description of conidial surface proteins	[323]
	Conidia, mycelium, whole cell proteome	TMT-LC-MS/MS	75	Proteomic under simulated Mars conditions	[352]
*Aspergillus niger*	Mycelium, whole cell proteome	TMT-LC-MS/MS	327	Protein characterization of an isolate from the International Space Station	[347]
		iTRAQ LC-MS/MS	1025	Characterization of the proteome under heat stress	[277]
*Aspergillus terreus spp.*	Conidia, whole cell proteome	LC-MS-QToF	Not indicated	Identification of proteins expressed under simulated deep-sea condition	[331]
*Aspergillus sydowii*	Conidia, whole cell proteome	LC-MS-QToF	Not indicated	Identification of proteins expressed under simulated deep-sea condition	[331]
*Aspergillus versicolor*	Conidia	2D-E, IgE-immunoblotting	20	Identification of allergens detected in sera from patients participating in a study about indoor exposure to molds	[262]
*Beauveria bassiana*	Secretome	Label-free nano-LC-MS/MS	50	Secretome screening under two diet regimes	[299]
*Botrytis cinerea*	Mycelium, whole cell proteome	iTRAQ LC-MS/MS	3816	Differential expression study of proteins following fungal treatment with the broad-spectrum agricultural antibiotic wuyiencin	[263]
		MALDI, LC-ESI-MS/MS	1431	Identification of target proteins and pathways of the antifungal peptide ETD151, homologous to insect defensin	[264]
	Secretome	2D-E, LC-MS/MS	56, 105	Description of the fungus secretome	[292]
		Nano-LC-MS/MS	1719	Comparative secretome analysis of wild type and non-virulent mutants	[293]
*Candida albicans*	Yeast cells, whole cell proteome	2D-E, MALDI-TOF/TOF	51	Identification of proteins regulated by fungal exposure to oleic acid from the curry leaf tree *Murraya koenigii*	[257]
		Label-free LC-MS/MS	1262	Quantitative proteomics of the response to osmotic stress.	[279]
	Chlamydospores	SWATH-MS	1177	Description of chlamydospores proteome	[280]
	Cell wall proteome	MALDI-TOF/TOF	46	Identification of surface exposed proteins.	[218]
			131	Analysis of surface exposed proteins under different growth conditions.	[315]
			50	Proteomic analysis of a pga1 null strain	[322]
*Candida glabrata*	Secretome	LC-MS/MS	119; 548	Secretome characterization of wild type and Cgyps1-11Δ mutant strain.	[301]
*Cladonia stellaris*	Thallus proteome	IEF	n.a.	Description of enzyme polymorphism	[375]
*Cladonia portentosa*	Thallus proteome	2D-E, MALDI-TOF, MALDI-TOF/TOF	45	Proteomic analysis of nitrogen stress effects	[382]
*Cladosporium cladosporioides*	Conidia, mycelium, whole cell proteome	TMT-LC-MS/MS	269	Proteomic under simulated Mars conditions	[352]
*Coccidioides posadasii*	Cell wall proteome	UHPLC-MS^E^	314	Detection of protein targets for broad-spectrum mycosis vaccines	[325]
*Cryomices antarcticus*	Whole cell proteome	2D-E	n.a.	Characterization of protein patterns under simulated Mars-like conditions	[340]
				Characterization of protein patterns under drought	[339]
*Debaryomyces hansenii*	Whole cell proteome	2D-E, MALDI-TOF/TOF	43	Proteomic changes under potassium starvation	[343]
*Doratomyces stemonitis*	Secretome	MALDI-TOF/TOF, Q-TOF LC-MS/MS	44	Secretome profiling	[364]
*Evernia prunastri*	Thallus proteome	2D-E, LC-ESI-MS/MS	5	Changes of the lichen proteome during exposure to constant concentrations of mercury	[378]
*Exophiala dermatitidis*	Mycelium, whole cell proteome	2D-DIGE, LC-ESI-MS/MS	32	Analysis of protein differential expression in response to temperature stress	[276]
*Exophiala jeanselmei*	Whole cell proteome	2D-E	n.a	Characterization of protein patterns in response to sub-optimal temperature	[338]
				Characterization of protein patterns under simulated Mars-like conditions	[340]
				Characterization of protein patterns under drought	[339]
*Fusarium oxysporum*	Secretome	Label-free LC-MS/MS	919	Detection of effector candidates regulated under simulated in-planta status	[296]
*Fusarium proliferatum*	Secretome	2D-E, MALDI-TOF MS	39	Investigation of pH-dependent changes in the fungus secretome	[295]
*Friedmanniomyces endolithicus*	Whole cell proteome	2D-E	n.a	Characterization of protein patterns in response to sub-optimal temperature	[338]
*Histoplasma capsulatum*	Extracellular vesicles	LC-MS/MS	206	Proteomic analysis of extracellular vesicles	[306]
			2000	Analysis of alterations in the extracellular vesicles proteome in response to different nutritional milieus	[309]
*Knufia chersonesos*	Secretome	LC-MS/MS	1730	Secretome screening for polyester degrading enzymes under exposure to PBAT.	[366]
*Knufia perforans*	Whole cell proteome	2D-E	n.a.	Characterization of protein patterns in response to sub-optimal temperature	[338]
				Characterization of protein patterns under simulated Mars-like conditions	[240]
				Characterization of protein patterns under drought	[339]
*Lobaria pulmonaria*	Proteome of the symbiotic consortium	1D-E, LC-ESI-MS/MS	463	Profiling of the metaproteome	[380]
			4405	Comparative omics to explore the lichen-associated microbiome	[380]
		1D-E, GeLC-MS/MS	6590	Profiling of the metaproteome	[381]
*Magnaporthe oryzae*	Mycelium and conidia, whole cell proteome	Label-free LC-MS/MS	355 and 559 N-glycosylation sites	Quantitative analysis of N-glycosylation regulation and elucidation of its role in development and pathogenesis of *M. oryzae*	[259]
		LC-MS/MS	5498	Comparative proteomic analysis between nitrogen supplemented and starved conditions	[278]
*Malbranchea cinnamomea*	Secretome	LC-MS/MS	53	Secretome screening for lignocellulolytic enzymes	[361]
Metarhizium anisopliae	Secretome	MudPIT-MS/MS	48	Analysis of the secretome induced by insect cuticle	[300]
*Metarhizium robertsii*	Mycelium and conidia, whole cell proteome	iTRAQ	2052	Comparative quantitative analysis of conidia and mycelium proteome	[266]
*Mycothermus thermophilus*	Secretome	Q-TOF LC/MS	240	Secretome screening for cellulases and hemicellulases	[362]
*Paracoccidioides sp.*	Yeast cells, whole cell proteome	2D-E, MALDI-MS/MS	135	Comparative 2D-E to analyse protein differential expression in response to zinc deprivation.	[270]
		2D-E, MALDI-MS/MS	179	Characterization of the oxidative stress response following short- and long-term H_2_O_2_-treatment.	[271]
		NanoUPLC-MS^E^	421	Investigation of the proteome response to carbon starvation.	[273]
		NanoUPLC-MS^E^	142	Investigation of the proteome response to nitrosative starvation.	[274]
*Paracoccidioides brasiliensis*	Yeast cells, whole cell proteome	NanoUPLC-MS^E^	538	Analysis of the proteome response of *P. brasiliensis* Pb18following interaction with alveolar macrophages primed with interferon gamma.	[255]
		2D-E, LC-MS/MS	11	Identification of proteins commonly overexpressed in highly virulent *P. brasiliensis* spp. complex isolates causing disseminated disease in a murine model of PCM	[256]
		Nano-ESI-UPLC-MS^E^	308	Characterization of the proteomic response to macrophage internalization.	[410]
	Secretome	LC-MS/MS	205; 260	Vesicle and vesicle-free extracellular proteome.	[283]
*Paracoccidioides lutzii*	Yeast cells, whole cell proteome	UPLC-MS^E^	303	Analysis of proteins differential expression during osmotic shock.	[272]
	Cell wall proteome	NanoUPLC-MS^E^	512	Comparative analysis of yeast and mycelium cell wall proteome	[320]
*Penicillium sp.*	Conidia, whole cell proteome	LC-MS-QToF	Not indicated	Identification of proteins expressed under simulated deep-sea condition	[331]
*Penicillium oxalicum*	Membrane and subcellular proteome	2D-E, MALDI-TOF/TOF	20	Screening of membrane and cytosolic proteome in response to the polycyclic aromatic hydrocarbon (PAH) anthracene	[369]
*Pestalotiopsis sp.*	Secretome	LC-MS/MS	209	Characterization of saline and non-saline secretomes	[360]
*Physcia adscendens*	Thallus proteome	2D-E, MALDI-TOF-MS	16	Proteomic analysis in response to cadmium stress	[372]
*Pyrenophora tere*	Secretome	LC-MS/MS	182	Secretome profiling	[297]
*Pyricularia oryzae*	Mycelium, whole cell proteome	LC−MS/MS combined with a high-efficiency succinyl-lysine antibody	714 and 2109 lysine succinylation sites	Succinyl-proteome profiling of Pyricularia oryzae and role of succinylation in general metabolism and infection	[260]
*Sclerotinia sclerotiorum*	Mycelium, whole cell proteome	1D-E, LC-MS/MS	1471	Proteomic profiling of *S. sclerotiorum* total proteome	[269]
*Sporotrichum thermophile*	Whole cell proteome	2D-E, LC-ESI-MS/MS	15	Proteomic profiling under the effect of ionic liquids	[346]
*Sporothrix brasiliensis*	Extracellular vesicles	2D-E, LC-MS/MS	63	Detection of EVs associated immunogenic components and virulence factors	[310]
*Sporothrix schenckii*	Extracellular vesicles	2D-E, LC-MS/MS	40	Detection of EVs associated immunogenic components and virulence factors	[310]
	Cell wall proteome	1D-E, LC-ESI-HDMS^E^	479	Proteomic analysis of the cell wall in response to oxidative stress	[321]
*Talaromyces marneffei*	Mycelium and conidia, whole cell proteome	2D-DIGE, MALDI-TOF MS	26	Differentially expressed proteins in yeast and mycelial phases	[267]
	Mycelium and conidia, secretome	2D-E, MALDI-TOF MS	12	Proteome profiling of the extracellular proteome	[268]
*Thermomyces lanuginosus*	Secretome	LC-MS/MS	74	Characterization of secretome during fungal growth on corn cobs	[363]
*Trichoderma harzianum*	Secretome	2D-E, MALDI-TOF MS	60	Analysis of the fungus secretome during growth on plant cell wall	[294]
*Trichoderma reesei*	Secretome	iTRAQ	636	Description of secretome composition in the wild type and in a hypercellulolytic mutant	[337]
*Umbilicaria mammulata*	Thallus proteome	IEF	n.a.	Intraspecific variability of isozymes	[374]
*Xanthoria parietina*	Thallus proteome	1D-E	n.a	Characterization of proteins involved in the lichen-algae interplay.	[373]
*Yarrowia lipolytica*	Whole cell proteome	2D-E, MALDI-TOF/TOF	38	Proteomic analysis of the response to environmental pH stimuli	[342]
*Zymoseptoria tritici*	Mycelium, whole cell proteome	1D-E, SCX, LC-MS/MS	6440	Comprehensive proteomic analysis of the proteome of *Z. tritici* during growth in nutrient-limiting and rich media and in vivo at a late stage of wheat infection	[258]
